# Bayesian-based noninvasive prenatal diagnosis of single-gene disorders

**DOI:** 10.1101/gr.235796.118

**Published:** 2019-03

**Authors:** Tom Rabinowitz, Avital Polsky, David Golan, Artem Danilevsky, Guy Shapira, Chen Raff, Lina Basel-Salmon, Reut Tomashov Matar, Noam Shomron

**Affiliations:** 1 Sackler Faculty of Medicine, Tel Aviv University, Tel Aviv, 6997801, Israel;; 2 Faculty of Industrial Engineering and Management, Technion, Haifa, 3200003, Israel;; 3 Raphael Recanati Genetic Institute, Rabin Medical Center, Beilinson Hospital, Petah Tikva, 4941494, Israel

## Abstract

In the last decade, noninvasive prenatal diagnosis (NIPD) has emerged as an effective procedure for early detection of inherited diseases during pregnancy. This technique is based on using cell-free DNA (cfDNA) and fetal cfDNA (cffDNA) in maternal blood, and hence, has minimal risk for the mother and fetus compared with invasive techniques. NIPD is currently used for identifying chromosomal abnormalities (in some instances) and for single-gene disorders (SGDs) of paternal origin. However, for SGDs of maternal origin, sensitivity poses a challenge that limits the testing to one genetic disorder at a time. Here, we present a Bayesian method for the NIPD of monogenic diseases that is independent of the mode of inheritance and parental origin. Furthermore, we show that accounting for differences in the length distribution of fetal- and maternal-derived cfDNA fragments results in increased accuracy. Our model is the first to predict inherited insertions–deletions (indels). The method described can serve as a general framework for the NIPD of SGDs; this will facilitate easy integration of further improvements. One such improvement that is presented in the current study is a machine learning model that corrects errors based on patterns found in previously processed data. Overall, we show that next-generation sequencing (NGS) can be used for the NIPD of a wide range of monogenic diseases, simultaneously. We believe that our study will lead to the achievement of a comprehensive NIPD for monogenic diseases.

Noninvasive prenatal diagnosis (NIPD) has become increasingly popular in the last few years. Typically, it is achieved by analyzing cell-free DNA (cfDNA) in the maternal plasma, which contains fetal cfDNA (cffDNA) derived from the placenta. Its main use is for identifying chromosomal abnormalities, for example, trisomy 21 (Lo et al. 2007; Fan et al. 2008). Other clinical applications are fetal sex determination (Hill et al. 2012; Lewis et al. 2012) and Rhesus D genotyping (Finning et al. 2004; Minon et al. 2008). NIPD of single-gene disorders (SGDs) is considered the next frontier in this field. Genetic diagnosis of SGDs is regularly achieved by using many clinical tools and methods. These range from the phenotypic description and a linkage analysis, through various laboratory tests, such as polymerase chain reaction (PCR) and DNA microarrays for known mutations, to Sanger sequencing for confirmation of results and next-generation sequencing (NGS) for a deeper investigation (Mahdieh and Rabbani 2013), usually using whole exome/genome sequencing (WES/WGS) (Isakov et al. 2013; Yang et al. 2013). Because the cost of WGS is still high and the implications of its results are less studied, WES, which covers ∼2%–3% of the genome and is less costly, remains more commonly used. WES of infants suspected of genetic disorders was recently shown to be more likely to affect medical care (Meng et al. 2017), and WES of DNA obtained by amniocentesis was reported to assist prenatal diagnosis in several cases (Mackie et al. 2014; Vora et al. 2017).

The application of NGS to the NIPD of monogenic diseases has already demonstrated feasibility, yet some improvements may still be possible. Identification of the paternally transmitted allele in cfDNA is considered to be straightforward (Fan et al. 2012; Kitzman et al. 2012) and is already used clinically for specific genes (Hill et al. 2015). However, maternally transmitted alleles pose a greater challenge, since in sites where the mother is heterozygous, both alleles are found in her plasma. The current solution, relative mutation dosage (RMD), is based on allelic imbalance, that is, a slightly higher amount of one allele when the fetus is homozygous. Unfortunately, due to the low amounts of cfDNA, and even lower amounts of cffDNA, such determination is restricted to ultra-accurate devices such as digital PCR (Lun et al. 2008). Moreover, when more than a few genomic sites are tested, this method becomes less feasible. NGS can be used as well, but requires very deep coverage and therefore, is still limited to targeted genomic loci (Lam et al. 2012).

For the aforementioned reasons, to date, only a few attempts to noninvasively genotype a fetus have been made (Lo et al. 2010; Fan et al. 2012; Kitzman et al. 2012; Chen et al. 2013; Chan et al. 2016; Snyder et al. 2013). To overcome the required deep coverage, most of these studies included haplotyping of one or both parents, similar to what is done in relative haplotype dosage (RHDO) analysis (Lo et al. 2010). However, high-throughput techniques for genome-wide haplotyping are still sparse, they require laborious procedures, and limit the resolution of the inferred inheritance (Snyder et al. 2015; Chan et al. 2016; Jenkins et al. 2018). Fan et al. (2012) also tried to use WES to provide deeper coverage and showed promising results. In their study, they managed to reconstruct a high percentage of the fetal exome, when using deep WES, 221× and 631×, in the second and third trimesters, respectively. In their work, stringent data filtering was applied before the analysis. In the latest attempt to genotype a fetus by Chan and coworkers, a 270× WGS was performed, and a sequential probability ratio test (SQRT) was applied per site in loci where the mother is heterozygous, with no haplotyping of the parents (Chan et al. 2016). This method was termed genome-wide relative allele dosage (GRAD), which is a genome-wide application of the RMD approach. Together with improvements such as accurate detection of de novo mutations, this study showed the highest accuracy achieved so far for site-by-site inheritance prediction, however, with some limitations. First, the sequenced sample was from a third trimester pregnancy, in which both the amount of cfDNA and the fraction of cffDNA within it are high. Second, the applied method does not utilize available information about the paternal inheritance. Third, it is not clear whether a sequential test has an advantage when genotyping a single position, since the information in this case is not cumulative. Fourth, no results were presented for loci for which both parents were heterozygous. Finally, in their study, accuracy was calculated from a relatively low number of only 6.5 × 10^5^ sites where the mother was heterozygous and the father was homozygous.

An approach that can assist in improving noninvasive fetal genotyping could rely on inherent differences in features of fetal and maternal cfDNA fragments. For example, fetal-derived fragments have generally been reported to be shorter (Chan et al. 2004; Fan et al. 2010), and the pattern of their size distribution indicates a relationship with nucleosome positioning (Lo et al. 2010; Yu et al. 2014). Attempts to utilize these size differences have been made, but this was done mainly for chromosomal abnormalities (Cirigliano et al. 2017; Sun et al. 2017), with a hard threshold set in order to enrich for cffDNA (Sillence 2016). However, since the two size distributions largely overlap, such a threshold could lead to loss of relevant information (Fan et al. 2010), a problem that can be addressed through more sophisticated use of the size distributions (Arbabi et al. 2016).

In this study, we present a novel framework for the NIPD of SGDs. We introduce the widely practiced concepts of NGS-based variation analysis to this field, because we suggest that it is a unique case of variant calling. We use a Bayesian algorithm that incorporates information of each DNA fragment separately and utilizes unique features of fetal-maternal origin, such as the fragment length. This is done using weights, rather than by setting a hard threshold, thus utilizing all fragments. Our method extends to small insertions and deletions (indels), and to loci for which both parents are heterozygous, thus supporting its generalizability. We developed *Hoobari*, the first software tool for noninvasive prenatal genotyping. *Hoobari* is straightforward, easy to use, and produces output that is compatible with existing tools for downstream analyses. *Hoobari*’s results can be further improved using a machine learning–based step that leverages previously analyzed data, similar to the existing variant recalibration algorithms. We demonstrate the ability of our model to resolve the diagnosis of SGDs using NGS; this will lead to a straightforward NIPD of a wide range of SGDs.

## Results

### Utilizing fragment sizes for fetal genotyping

**Figure 1.**
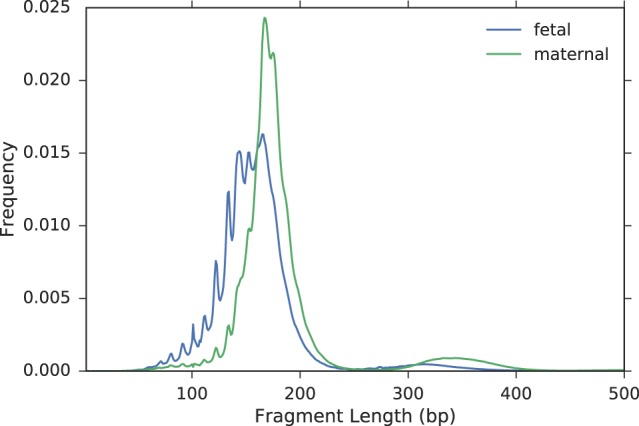
Length distributions of fetal and maternal fragments in family G1.

To use the inherent properties of fetal and maternal cfDNA, we first attempted to determine whether the differences in size between these cfDNA fragments can improve genotyping accuracy. We were specifically interested in SNPs where the mother is heterozygous, but also tested the same model over SNPs where only the father is heterozygous, to demonstrate generalizability to all loci in the genome. In loci where the mother is heterozygous, both alleles are present in the plasma, making it impossible to determine whether a fragment is fetal or maternal. In our algorithm, each fragment has a certain probability of being fetal, depending on its length. To this end, we measured two empirical length distributions, fetal and maternal, using sites at which the parents are homozygous for different alleles (Fig. 1; Supplemental Fig. S1). In these sites, a cfDNA fragment that presents the paternal allele is considered to be fetal-derived. Next, we calculated the total fetal fraction, which is the fraction of cffDNA within all maternal cfDNA, as previously described (Chan et al. 2016). Then, we calculated a fetal fraction for each fragment size, using all fragments with the same length. During the genotyping step, each read was assigned the corresponding per-size fetal fraction. Accordingly, shorter fragments generally received a higher probability of being fetal, and a stringent size threshold could be avoided.

**Figure 2.**
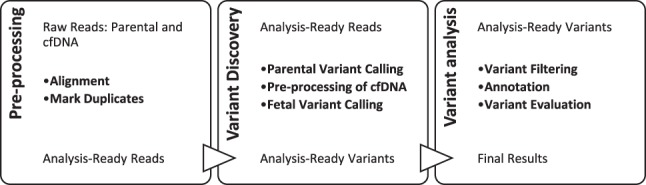
*Hoobari*’s pipeline for noninvasive prenatal variant calling.

We used the sequencing data of the parents and cfDNA as input for our pipeline (Fig. 2). This workflow differs from regular variant calling in two main aspects: (1) The prior probabilities can be calculated using the existing parental sequencing data; therefore, an initial genotyping of the parents is required; and (2) cfDNA is an unbalanced mixture of two similar genomes and requires a dedicated algorithm for calculating the likelihoods in the Bayesian model. This algorithm uses the aforementioned calculation of the per-size fetal fraction (Methods).

*Hoobari* returned three posterior probabilities, one for each possible fetal genotype: homozygous to the reference allele (0/0), heterozygous (0/1), and homozygous to the alternate allele (1/1). The predicted genotype in each site is the one with the highest posterior probability. Fetal variants that were found using pure fetal tissue, such as amniotic fluid, chorionic villi, and umbilical cord blood were used as the ground truth.

Two main factors were formerly shown to affect the accuracy of NIPD: the fetal fraction and the cfDNA sequencing depth. We tested our algorithm on whole-genome data of four family trios with different fetal fraction values, which were sequenced to different depths of coverage in two previous studies (Kitzman et al. 2012; Chan et al. 2016). In the first two families, G1 and G2, the sequencing depth of the cfDNA and the fetal fraction were very high (Table 1), and their sequencing was performed using a PCR-free library preparation protocol, which is considered to be more accurate. These families were selected to measure our model's performance with the best sequencing and biological settings available. Families G3 and G4, in which the depth and the fetal fraction were considerably lower, were selected to estimate our model's performance in more challenging sequencing settings, but with a fetal fraction that is more clinically relevant. We compared our results to those obtained from methods that do not account for size distribution: (1) a fixed fetal fraction-based version of *Hoobari* (Methods); and (2) GRAD analysis, which, in addition, does not utilize the paternal information and is based on a sequential statistical test.

**Table 1.**
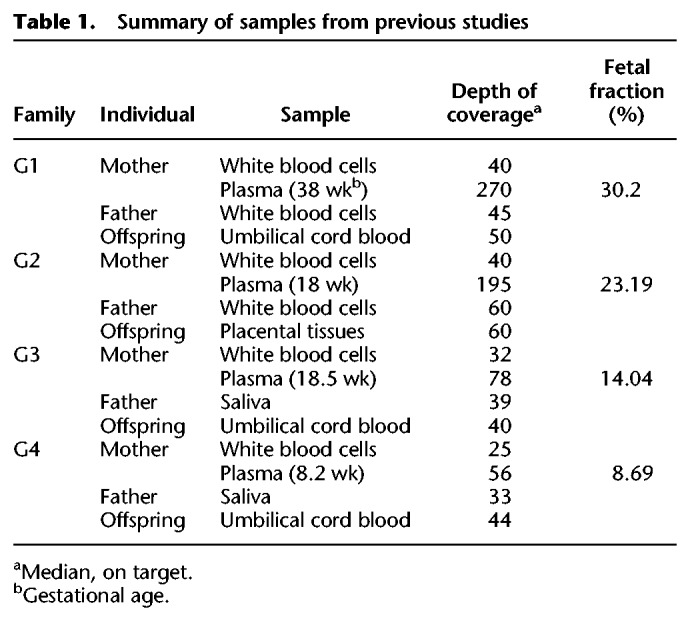
Summary of samples from previous studies

**Figure 3.**
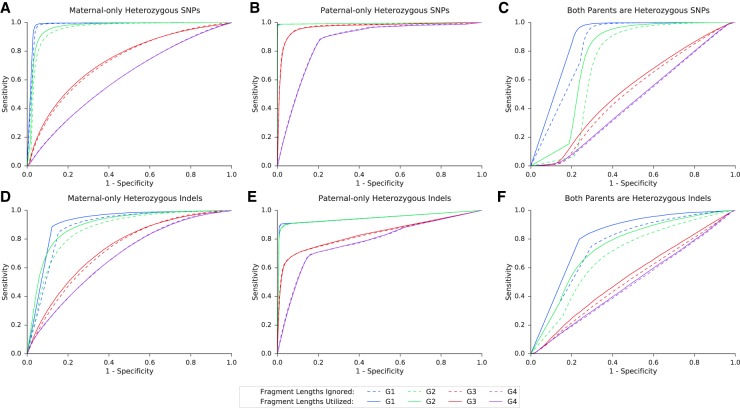
Performance of the model over SNPs and indels in families G1–G4. Presented in the ROC curves are results for families G1–G4, in three categories of either SNPs (*A*–*C*) or indels (*D*–*F*), as described. In positions where only one parent was heterozygous, we examined whether the shared allele was transmitted to the fetus (based on the true fetal genotype). If both parents were heterozygous, we examined whether the true fetal genotype was heterozygous or homozygous. The corresponding AUC and accuracy values are shown in Table 2.

For each family we calculated the area under the receiver operating characteristic curve (ROC-AUC) and the accuracy (Table 2; Fig. 3A–C). Each tested case was divided into three categories of loci using the parental genotypes: maternal-only heterozygous (the father was homozygous), paternal-only heterozygous (the mother was homozygous), and double-heterozygous, in which both parents were heterozygous.

**Table 2.**
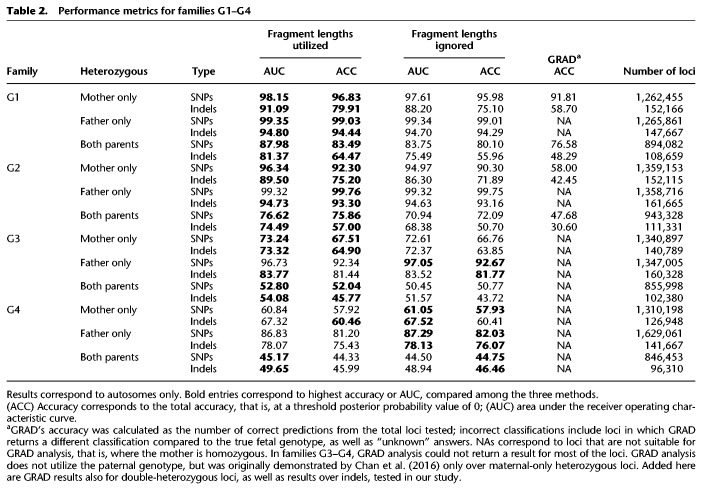
Performance metrics for families G1–G4

In all families, the accuracy was the highest among paternal-only heterozygous loci, followed by maternal-only heterozygous, and last, double-heterozygous loci. However, for a locus in one of the first two categories, the baseline accuracy is 0.5 since the outcome is either homozygous or heterozygous; whereas in a double-heterozygous locus the baseline is 0.33 since all three genotypes are possible. The utilization of fragment length information improved both the AUC and the accuracy. We compared our results to the original study in which G1–G2 were sequenced and analyzed using GRAD analysis (Chan et al. 2016). In that study, GRAD was tested over maternal-only heterozygous SNPs and not on double-heterozygous SNPs. The authors reported 610,084 correct predictions of 656,676 loci in family G1 (92.9%) and ∼511,112/775,456 in family G2 (65.9%). However, an individual is expected to have ∼3 million heterozygous SNPs in the genome, and ∼1.3 million maternal-only heterozygous SNPs are expected to be found (Kitzman et al. 2012; Li 2014). To test GRAD over the complete set of loci, we applied it on the variant set produced by *Hoobari*’s pipeline. *Hoobari* outperformed GRAD in all groups of positions and all families (Table 2). To demonstrate filtering of *Hoobari*’s results, we applied basic criteria at maternal-only heterozygous loci: a cfDNA depth of 100–1000 and posterior probability >0.99. This enabled achieving accuracies of 1,194,916/1,221,304 (97.84%) and 954,980/980,983 (97.35%) in families G1 and G2, respectively, thus further improving our results while maintaining a higher number of loci than the reported GRAD results. Families G3 and G4 showed considerably lower prediction results and the contribution of the information on fragment length was less consistent. We achieved 67.5% using site-by-site prediction of maternal-only heterozygous loci in family G3, slightly higher than the 64.4% achieved in the original study (Kitzman et al. 2012). These results support the explanation of Kitzman et al. (2012) of insufficient data to achieve confident calls within these samples. Therefore, we focused on families that were sequenced to a higher depth.

### Noninvasive prenatal indel calling

Next, we tested the performance of our algorithm over indels, using the same data of the aforementioned families. We used the same categories of loci and tested the utilization of fragment length information. Among the families at different categories, the results were similar to those described for SNPs, but with lower accuracy (Fig. 3D–F). The decreased accuracy was prominent across the maternal- and double-heterozygous loci, and mild across the paternal-heterozygous category. Utilization of the fragment lengths resulted in major improvement across the maternal- and double-heterozygous loci, and a smaller improvement in the paternal-heterozygous group. This effect was less consistent for families G3–G4.

### Subsampling of the fetal fraction and sequencing depth

**Figure 4.**
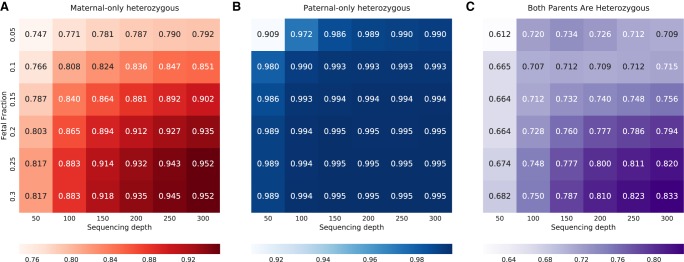
Performance of the model in SNPs, with different depths and fetal fractions. Heat maps *A*–*C* present the accuracy as a function of both the median sequencing depth and the fetal fraction, at the three categories of loci.

Our results confirm that fetal fraction and sequencing depth are important factors that affect the accuracy of our model. Because the fetal fraction in the first trimester is low, we aimed to examine the robustness of our model at low fetal fractions with high sequencing depth. However, the sequencing depth and fetal fraction in the previously sequenced families were either both low or both high. Therefore, we used family G1 to simulate 36 cfDNA samples, with a large range of values of fetal fraction and sequencing depth (Methods). At the highest fetal fraction with the greatest depth, results showed high accuracy for each loci category: 94.5% for maternal-only heterozygous loci; 98.6% for paternal-only heterozygous loci; and 82.3% for loci where both parents were heterozygous (Fig. 4). For fetal fraction values that are more common in the first trimester, that is, 10%–15%, accuracy values at the greatest sequencing depth were ∼87.1%, 98.5%, and 72.7% for the preceding categories, respectively. Results were achieved prior to any parental- and true-fetal-based filtering of loci. The simulations also revealed a pattern of decreasing accuracy over the three site categories. Maternal-only heterozygous loci depend on both the sequencing depth and the fetal fraction. Paternal-only heterozygous loci are robust even at low fetal fractions and depths, but not under both conditions simultaneously. Double-heterozygous loci are sensitive to both factors, and somewhat more to the sequencing depth. Similar results were presented on indels (Supplemental Fig. S2), except for an improved accuracy at double-heterozygous indels, with a fetal fraction <0.2 at lower depths.

### Analysis of first trimester cases

After better defining the sequencing settings required to achieve high accuracy, we attempted to test our model over first trimester families, which were sequenced to a high coverage. Three additional families were sequenced using different methods that aimed to achieve a high depth of coverage (Table 3). In two families, E1 and E2, the cfDNA sample was sequenced using WES, that is, ∼2%–3% of the genome (Methods). In family E2, the parents and the chorionic villus sample were sequenced using WGS as an attempt to decrease the rate of errors that are not related to the cfDNA. Family G5 was sequenced using deep WGS with a PCR-free library preparation protocol in order to avoid errors that are related to amplification or WES. However, three cycles of PCR were required after the library preparation step. The cfDNA sequencing depths and fetal fraction values for families E1–E2 and G5 are presented in Table 3.

**Table 3.**
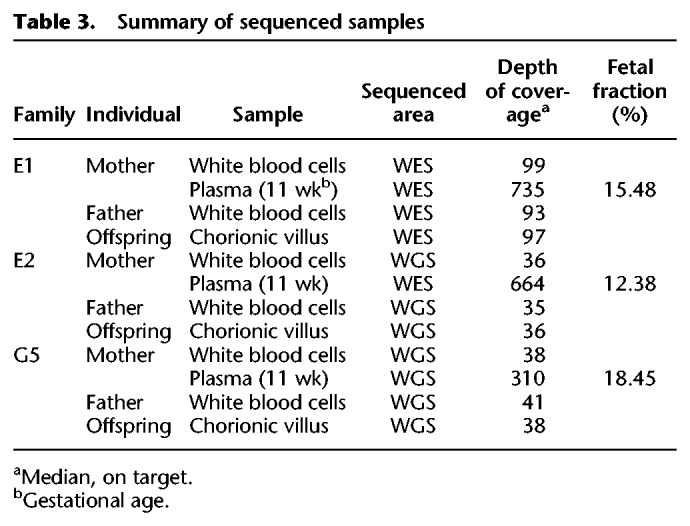
Summary of sequenced samples

**Figure 5.**
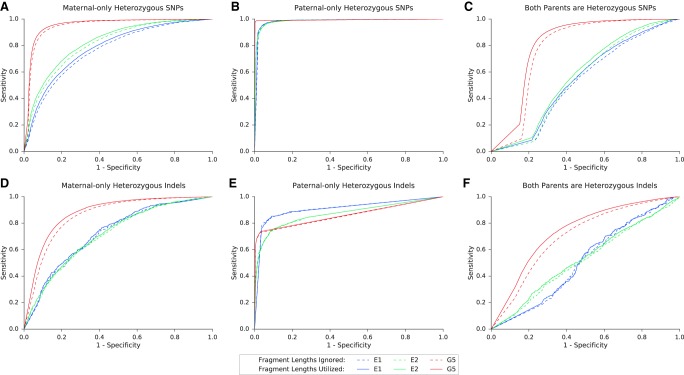
Performance of the model over SNPs and indels in families G5, E1, and E2. Presented in the ROC curves are results for families G5, E1, and E2 in three categories of either SNPs (*A*–*C*) or indels (*D*–*F*), as described. In positions where only one parent was heterozygous, we examined whether the shared allele was transmitted to the fetus (based on the true fetal genotype). If both parents were heterozygous, we examined whether the true fetal genotype was heterozygous or homozygous. The corresponding AUC and accuracy values are shown in Table 4.

As done earlier, the algorithm was tested over SNPs and indels in maternal-only heterozygous loci, paternal-only heterozygous loci, and double-heterozygous loci (Table 4; Fig. 5). The overall accuracy was limited in the WES-sequenced families E1–E2, and this was especially noticeable in double-heterozygous sites. Family G5 results were considerably better at all settings and aspects. Results again showed improvement subsequent to the addition of the fragment length information in all comparisons.

**Table 4.**
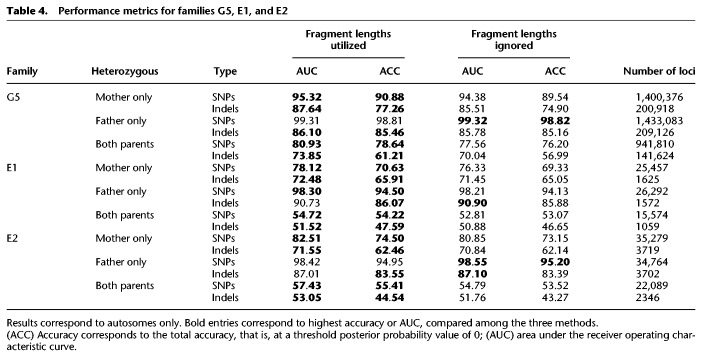
Performance metrics for families G5, E1, and E2

Among the predicted SNPs, three deleterious mutations were examined. In family E1, the parents were carriers of a mutation in the *SLC26A3* gene, causing congenital chloride diarrhea, an autosomal recessive (AR) condition. In family G5, two mutations were detected, for which both parents were carriers: one mutation in *PCCA* causing propionic acidemia, and another mutation in *FKBP10* causing osteogenesis imperfecta; both are AR conditions. The parents in family E2 were carriers of a structural mutation in *SMN1*, which was not in the scope of our study, and were not carriers of either of the other mutations. Therefore, it served as a negative control. Families E1 and G3 also served as negative controls of each other. We successfully predicted that the fetus in family E1 is homozygous to the mutant allele, a result that matched the WES of the chorionic villus sampling (CVS) and was further validated using Sanger sequencing (Supplemental Fig. S3). In the relevant site, the posterior probability of the predicted genotype increased from 56.5% to 61.7% when fragment length information was utilized. In family G5, we successfully predicted the fetus to be a carrier of the *PCCA* mutation, as predicted by the CVS's WGS and Sanger sequencing results (Supplemental Fig. S3). The posterior probability of this result increased from 83.9% to 99.4% when the fragment lengths were used. We were not able to successfully predict the genotype in *FKBP10*; although Sanger and WES sequencing showed the fetus to be a carrier, our results showed homozygosity to the mutant allele. Utilizing the fragment lengths resulted in lower probability of this false positive result, 84.6% compared to 96.0%, while the probability of heterozygosity increased from 4.0% to 15.4%. In all cases, none of the negative controls were predicted as carriers or as homozygous to the tested mutations. GRAD analysis correctly predicted the mutation in *FKBP10*, and was not able to return a prediction for the other mutations.

### Machine learning–based variant probability recalibration

Popular variant analysis pipelines include a step of variant recalibration, in which the results of previously analyzed data are used for finding and correcting error patterns in a new sample (Van der Auwera et al. 2013). During this step, a machine learning model is trained based on features that are not directly modeled by the variant caller and that typically include its sequencing depth, strand balance, and other information. The output is a score that corresponds to each variant, which better represents the sensitivity, specificity and accuracy. This score can then be used for filtering, depending on the desired level of confidence. Current variant recalibration methods cannot be fitted to the unique cfDNA case in a straightforward manner, predominantly because they do not utilize important parental information that is available in the context of noninvasive fetal genotyping. Therefore, we sought to demonstrate how a new model can adjust *Hoobari*’s results in a way that would improve the accuracy and ROC-AUC.

For the first training set, we chose family G1, for whom the sequencing depth and fetal fraction were the highest. Family G2 was randomly divided: 75% of the variants were used as a validation set throughout the training process, and the remaining 25% served as a test set at the end of the training. To demonstrate that our model can be generalized to other data sets, we used family G5 as a second test set. The features in our model were taken from the metadata that are available when genotyping the parents and the cfDNA, as well as *Hoobari*’s classifications and probabilistic results for each possible genotype (Supplemental Table S1). Various models were compared, and the Random Forest algorithm was found to yield the best results in terms of accuracy, running time, and ROC-AUC (Methods; Supplemental Table S2). At first, the model for each category of variants was trained based on variants from the same group only, for example, a model for maternal-only heterozygous SNPs was trained over maternal-only heterozygous SNPs. We found that in the categories of maternal-only and double-heterozygous SNPs, this suffices to improve the accuracy and AUC. However, in paternal-only heterozygous SNPs, improvement was achieved only when SNPs from all three categories were used for training. This may be due to the low error rate within this group. To deal with the low number of indels in the training set, we trained their models on both SNPs and indels within the same category.

**Figure 6.**
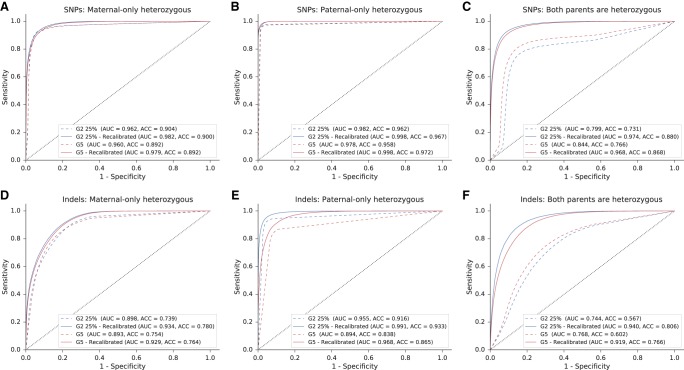
Results of the machine learning–based variant recalibration step. ROC curves of the two test sets, before and after the machine learning–based variant recalibration step, are presented for three categories of either SNPs (*A*–*C*) or indels (*D*–*F*), as described. For each curve, the microaveraged area under the curve (AUC) and the total accuracy (ACC) are presented. In contrast to the results presented in Figures 3–5, no filtering was applied to parental and true fetal variants prior to this analysis.

The trained models were then tested once on the remaining 25% of G2. The same model architecture was trained again on the combined data of families G1 and G2, and then tested once on family G5. The results showed that previous data can indeed be applied to improve performance for a new family. The AUC was improved in all categories of loci, and accuracy was improved in almost all cases (Fig. 6). Prominent improvement was seen in loci where both parents were heterozygous, a condition that originally presented low accuracy. This opens the possibility of performing post-genotyping filtering of loci using the probability output of the recalibration step rather than setting stringent thresholds for specific features (Supplemental Fig. S4). As an example, we checked the results when filtering out all variants with a recalibration score <0.7 and found that the accuracy in family G5 improved to 98.3%–99.8% over SNPs, from a total of 722,630 remaining maternal-only heterozygous; 1,358,503 paternal-only heterozygous; and 358,114 double-heterozygous SNPs. Indel prediction accuracy improved to 94%–97%, from 42,726 maternal-only heterozygous; 142,577 paternal-only heterozygous; and 20,388 double-heterozygous indels. Last, we found that *Hoobari*-derived features, such as the posterior probabilities, the likelihoods, and the predicted genotype have the greatest importance, followed by features related to the allelic balance in the cfDNA (Supplemental Table S3).

## Discussion

In this study, we performed upgraded noninvasive fetal genotyping, using a novel approach and an improved algorithm, which was implemented as *Hoobari*, the first software tool for noninvasive prenatal variant calling. We showed that certain characteristics, such as the size differences between maternal- and fetal-derived fragments improve cfDNA-based fetal genotyping. State-of-the-art results were achieved at sites where the mother is heterozygous, which currently pose the greatest identification challenge.

Using the same algorithm, we also predicted inherited indels, although with lower accuracy. Adding the fragment size information and recalibrating the results using machine learning improved these results, but the accuracy remained lower than that achieved for SNPs. Indels are the second most common type of variants and can be deleterious, especially when they affect the reading frame (Mullaney et al. 2010; Neuman et al. 2013). Compared to SNPs, which are much more common and easier to predict, indel calling in individuals still lags behind, and detection methods display considerable discrepancy (Jiang et al. 2015; Hwang et al. 2016). Reasons for this may be the higher rate of alignment errors, and the larger number of possible alleles in the parents, which all lead to lower prediction confidence. Nevertheless, any solution for NIPD of SGDs will have to address this issue, and our study presents one way of accomplishing it.

We suggest that a Bayesian approach, which is the core of this study, is most suitable for our task. One advantage of this approach is that it is modular, in the sense that it enables adding available information. In our case, we only used fragment size information for the probabilistic separation of fetal- and maternal-derived reads; however, other features might help in asserting the probability of each read being fetal. These could be other characteristics of cffDNA, which have been recently described. For example, it has been shown that fetal-derived fragments tend to arrive from clusters of preferred ending positions (Chan et al. 2016). Haplotype information can also be integrated into the model when it becomes more widely available. This will enable determining the origin of each fragment with greater confidence. These features can be integrated into a more sophisticated classifier that performs a probabilistic separation of the reads.

Filtering the called variants is based on continuous parameters that require a cutoff value; however, determining this value is usually not clear. To avoid arbitrary thresholds, we focused on describing a distribution of genotyping probabilities. We aim to predict the fetal genotype at all parental sites that passed a very lenient set of filters, to achieve high sensitivity. Only at this point are different annotations, statistical tests, and machine learning recalibration methods applied, so that the low confidence results can be filtered out and the specificity improved. This consistency with the accepted process of variant calling is another advantage of the Bayesian approach, because the posterior probabilities can be used as a filtering parameter.

In our method, DNA is collected, shifted, and sequenced in a straightforward manner, without haplotype reconstruction or other unique protocols. This demonstrates that a simultaneous NIPD of a large range of SGDs is feasible with available technology. We showed that this is possible at 11 weeks of gestation by performing the deepest first trimester WGS of cffDNA to date. However, WES resulted in lower accuracy than PCR-free WGS, even with a greater sequencing depth. These results can be explained by the amplification steps required for both the WES library preparation and low-input protocols; these affected the length distributions (Supplemental Fig. S1) and increased the number of sequencing errors. WES was previously shown to be less powerful than WGS, even for exome variants (Belkadi et al. 2015). Nevertheless, the probabilistic scaling used in our model ensures that even when using WES, a proportion of the sites will be genotyped with high confidence. Just as with regular variant discovery, these sites can be used in downstream analysis to identify rare variants that are yet to be discovered. Moreover, if a more accurate WES or any other targeted NGS technique is used, accuracy might improve without having to rely on deep WGS. While conducting our study, we noticed that the fragment length frequencies are similar across pregnancies. Consequently, the fetal fraction at each fragment length can be theoretically approximated by using only these frequencies and the total fetal fraction. Because the total fetal fraction itself can be approximated using a relatively small number of loci, we suggest that our method is scalable, that is, can be applied over small or large sequenced regions. Finally, owing to its low cost, with WES, it is currently more feasible to create a large data set of family trios that can be further analyzed to improve NIPT of SGDs.

Our study and method have some limitations. First, some types of sites are not yet supported. These include variant sites that are not biallelic and de novo mutations. For other types of genomic sites, such as those where only the father is heterozygous, our algorithm applies as well; however, optimization is still needed. Second, we intentionally avoid haplotyping of the parents; yet it might be required for assessing compound heterozygosity. Third, our method currently requires a specific variant calling software, and was tested using an off-the-shelf read alignment method. The combination with alternative methods, as well as careful realignment and local reassembly, might improve its performance. An example of a consequence of such is the high number of false positive indel calls that was previously shown in FreeBayes (https://github.com/ekg/freebayes) and that might be lower in other variant callers (Cornish and Guda 2015). DNA extraction, library preparation and sequencing methods should be further optimized as well. Family G5, for example, was sequenced using a PCR-free library preparation protocol, yet three PCR cycles were still required prior to sequencing. This raises the possibility of added bias, as implied by the length distribution (Supplemental Fig. S1). Fourth, our method was tested on seven trios from three to four data sets with varying sequencing settings, only four of them were first trimester cases. Although this is more than previous methods, which tested on only one to two families from one data set, more families should be sequenced to further evaluate our model. Moreover, even fewer families were assessed in the recalibration step; this could reduce the generalizability of the method. The recalibration step is, however, a proof-of-concept for the ability to improve *Hoobari*’s results using external data, that is from other families, similar to other variant recalibration methods. We believe that with time, accumulating data will improve this step and further demonstrate generalizability. Lastly, we have not addressed the major ethical concerns related to the subject. The increasing availability of genome sequencing has already given rise to many ethical disputes. Turning prenatal WES/WGS into a simple and available test requires that it be used responsibly. For instance, the results obtained need to be filtered, and only sites of interest and well-described variants should be used so as to prevent incidental findings and variants of unknown significance. In general, maintaining high accuracy in smaller areas of the genome, such as gene panels, could contribute to the clinical relevance of the method.

In summary, we present a general approach for fetal variant detection, in which we used cfDNA and parental sequencing data together with a novel algorithm. These concepts can be extended to other fields, such as cancer detection and monitoring, using circulating tumor DNA (Mouliere et al. 2018). In this study we laid the infrastructure for noninvasive prenatal variant calling; we foresee a future in which sequencing of the fetal genome from maternal blood will be commonly performed for diagnosing diseases caused by single mutations.

## Methods

### Fetal fraction and depth reduction

We define *f* as the observed fetal fraction at a given variant site, *f*_TOTAL_ as the mean *f* value over all sites, and *d* as the desired fetal fraction. Of the fetal reads covering each site, 1 − *d*/*f* should become maternal-like by assigning them a different fragment size, and in some instances, also a different represented allele (*d* < *f*, otherwise we use *f*_TOTAL_) (Supplemental Fig. S5). At sites where a fetal-specific allele could be recognized, we randomly discarded *N* = 1 − *d*/*f* of the reads presenting the fetal allele, and *N* was rounded by ceiling or floor functions randomly. Then, we sampled *N* reads presenting the shared allele and changed their lengths to values from the maternal length distribution. The probability of sampling each observed read length corresponded to its frequency within the fetal length distribution. The probability of assigning each new length corresponded to its frequency within the maternal length distribution. This enabled us to increase the rate of maternal-like fragment lengths within the group of shared allele reads. Finally, we generated *N* more reads presenting the shared allele and assigned them with lengths from the maternal length distribution. Again, the probability of assigning each length corresponded to its frequency in the maternal distribution. At positions in which the fetal-specific allele could not be recognized (where the mother is heterozygous), we sampled *d*/*f* of the reads using the fetal length distribution and assigned them with lengths from the maternal distribution. To down-sample the depth, the coding region of family G1 was used, and its median depth was calculated. To calculate the proportion of reads to sample, the desired median depth was divided by the measured median depth. For each loci, reads were randomly sampled by this proportion.

### Preprocessing of cell-free DNA data

FreeBayes was run on the cfDNA sample only at variant sites that were identified in the parental genomes. Using *Hoobari*, the allele that was observed by each read, together with the read insert-size, was saved in a separate database.

### Noninvasive fetal variant calling

*Hoobari* was run using the parental variants and the cfDNA preprocessing results database as input. The output was a variant call format (VCF) file.

### Bayesian noninvasive genotyping

At each site of interest, a Bayesian approach was applied. For each possible fetal genotype,

$$P(G|\mathrm{data})=\frac{P(\mathrm{data}|G)P(G)}{\sum_{i=1}^{n}P(\mathrm{data}|G_{i})P(G_{i})},$$

where *G* is the fetal genotype; and *G*_*i*_ is the *i*th possible fetal genotype of *n* possibilities. For biallelic variants, it would be either homozygous for the reference allele (AA), heterozygous (Aa), or homozygous for the alternate allele (aa). The prior probability of each genotype is denoted as *P*(*G*) and is calculated by Mendelian laws. The data are the reads that cover a site and *P*(data|*G*) is the likelihood function, which is a product of the likelihood of each read-observation:

$$P(\mathrm{data}|G)=\overset{m}{\underset{\;j=1}{\prod }}P(r_{j}\;|G,G_{M},f)=\overset{m}{\underset{\;j=1}{\prod }}(P(r_{j}|\mathrm{fet})P(\mathrm{fet})+P(r_{j}|\mathrm{mat})P(\mathrm{mat})).$$

The likelihood of a read *r*_*j*_ depends on the fetal genotype and is calculated using the maternal genotype and the fetal fraction. *P*(*r*_*j*_|*fet*) and *P*(*r*_*j*_|*mat*) are the probabilities of a read-observation that supports a certain allele, given that the read is fetal and maternal, respectively. This depends on the tested fetal genotype *G*_*i*_, the maternal genotype *G*_*M*_, and the observed allele. *P*(fet) and *P*(mat) are the probabilities of observing a fetal or maternal read based only on the fetal fraction, regardless of the allele that it supports. For example, if the mother is heterozygous, the fetus is homozygous to allele *a*, the fetal fraction is *f*, and the observed read supports allele A; then the calculation is

$$P(r_{j}=A\;|G=aa,\;G_{M}=Aa,f)=0\cdot f+0.5\cdot (1-f),$$

since none of the fetal reads and half the maternal reads are expected to support allele A.

To utilize the size differences between fetal and maternal fragments, a fragment length-dependent fetal fraction was used with each observed read. The length-dependent fetal fractions were calculated by first grouping the cfDNA reads by their fragment lengths, then calculating the fetal fraction per group, similar to the calculation described in the Results section. Eventually, if the fragment size (*r*_TLEN_) of a read is 140 bp, then

$$P(r_{j}=A\;|G=aa,G_{\mathrm{mother}}=Aa,r_{\mathrm{TLEN}}=140)=0\cdot f_{140}+0.5\cdot (1-f_{140}).$$

Since the peak of the fetal length distribution is at 140 bp (Supplemental Fig. S1), it is expected that *f*_140_ > *f*. The peak of the maternal length distribution is at 166 bp; therefore *f*_166_ < *f*. For reads that were not properly paired or have a fragment size of >500, the total fetal fraction *f* was used.

### Ethics statement

All methods were performed in accordance with the relevant guidelines and regulations of the Institutional Review Board (IRB). IRB request 0825-RMC was submitted and approved under the national reference number 920160014.

### Supplemental methods

Processing of data sequenced in this study is covered in the Supplemental Methods; this includes sample collection, DNA extraction, library preparation, sequencing, alignment, and variant calling of the parents and fetus. Also included are methods used in the machine learning–based variant recalibration step.

## Data access

The sequence data from this study have been submitted to the NCBI database of Genotypes and Phenotypes (dbGaP; http://www.ncbi.nlm.nih.gov/dbgap) under accession number phs001659.v1.p1. *Hoobari* software source code is available at GitHub (https://github.com/nshomron/hoobari) and as Supplemental Code.

## Supplementary Material

Supplemental Material
