# Supplementary material for: Bayesian-based noninvasive prenatal diagnosis of single-gene disorders
Source: Genome Res. 2019 Mar;29(3):428–38. doi: 10.1101/gr.235796.118 (PMC6396420; doi:10.1101/gr.235796.118)
Supplement: Supplemental Material [file supp_29_3_428__index.html]

Bayesian-based noninvasive prenatal diagnosis of single-gene disorders — Bayesian-based noninvasive prenatal diagnosis of single-gene disorders — Supplemental Material 

# Bayesian-based noninvasive prenatal diagnosis of single-gene disorders

## Supplemental Material

- Supplemental\_Fig\_S1.pdf
- Supplemental\_Fig\_S2.pdf
- Supplemental\_Fig\_S3.pdf
- Supplemental\_Fig\_S4.pdf
- Supplemental\_Fig\_S5.pdf
- Supplemental\_Code.pdf
- Supplemental\_Table\_S1.xlsx
- Supplemental\_Table\_S2.xlsx
- Supplemental\_Table\_S3.xlsx
- Supplemental\_Methods.pdf
