# Supplementary material for: Bayesian-based noninvasive prenatal diagnosis of single-gene disorders
Source: Genome Res. 2019 Mar;29(3):428–38. doi: 10.1101/gr.235796.118 (PMC6396420; doi:10.1101/gr.235796.118)
Supplement: Supplemental Material [file supp_gr.235796.118_Supplemental_Fig_S1.pdf]

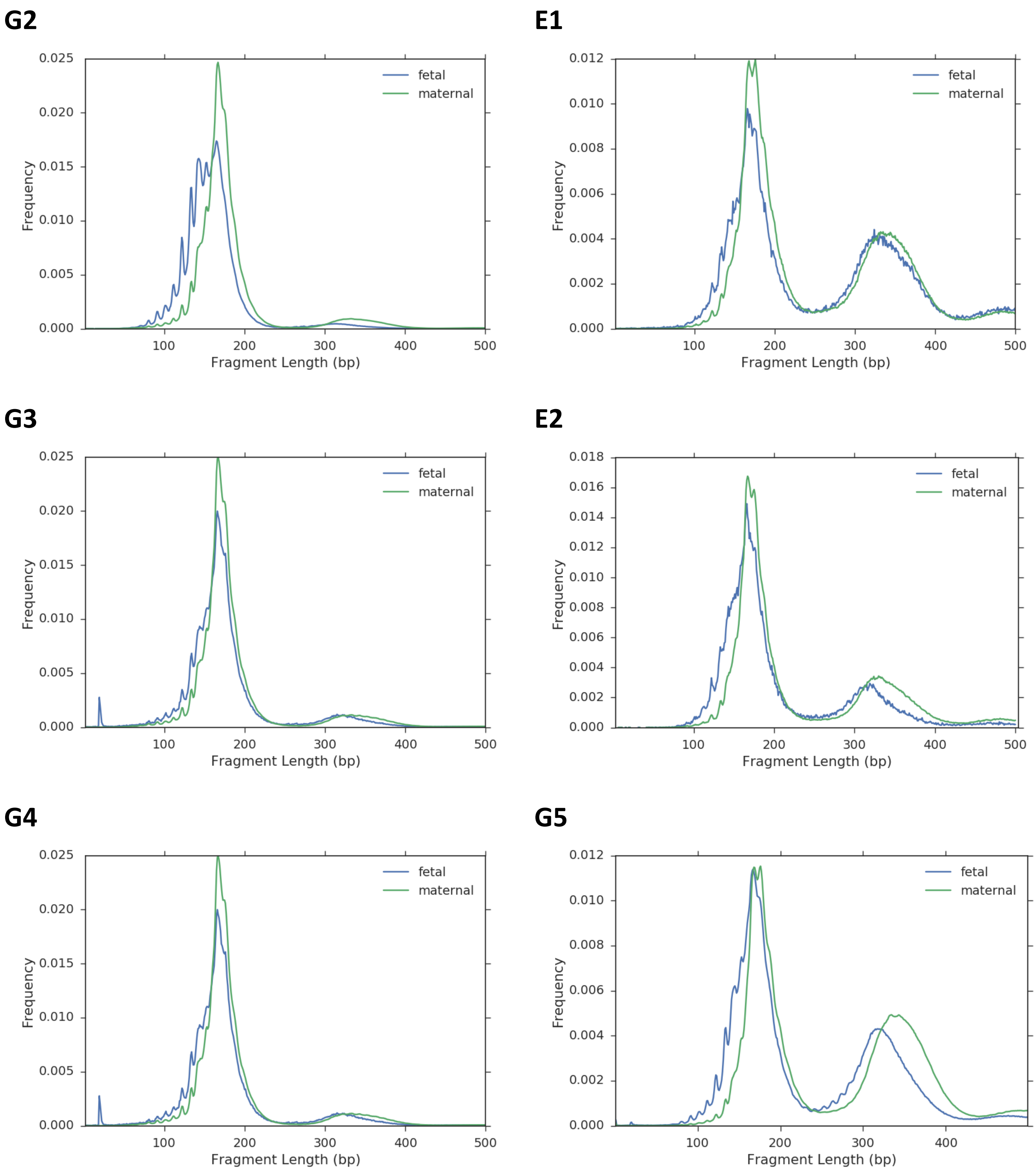

Supplemental Figure S1. Cell-free DNA fragment length distributions.

Presented are fetal and maternal fragment length distributions for families included in this study (family G1's distribution is shown in Figure 2 of the manuscript).
