# Supplementary material for: Bayesian-based noninvasive prenatal diagnosis of single-gene disorders
Source: Genome Res. 2019 Mar;29(3):428–38. doi: 10.1101/gr.235796.118 (PMC6396420; doi:10.1101/gr.235796.118)
Supplement: Supplemental Material [file supp_gr.235796.118_Supplemental_Fig_S2.pdf]

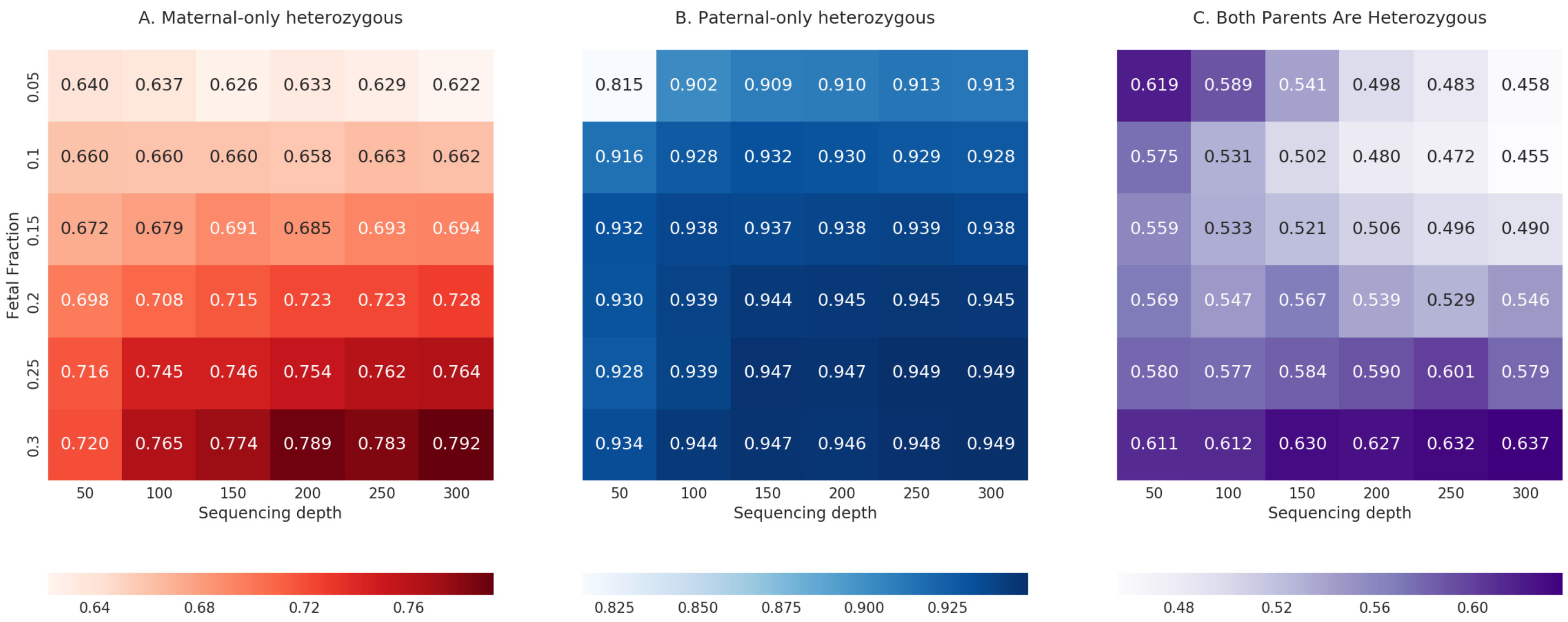

Supplemental Figure S2. Performance of the model in indels with different depths and fetal fractions.

Heat maps A-C present the indel prediction accuracy as a function of both the sequencing depth and the fetal fraction, at the three categories of loci that were described in the manuscript.
