# Supplementary material for: Bayesian-based noninvasive prenatal diagnosis of single-gene disorders
Source: Genome Res. 2019 Mar;29(3):428–38. doi: 10.1101/gr.235796.118 (PMC6396420; doi:10.1101/gr.235796.118)
Supplement: Supplemental Material [file supp_gr.235796.118_Supplemental_Fig_S3.pdf]

E1

SLC26A3

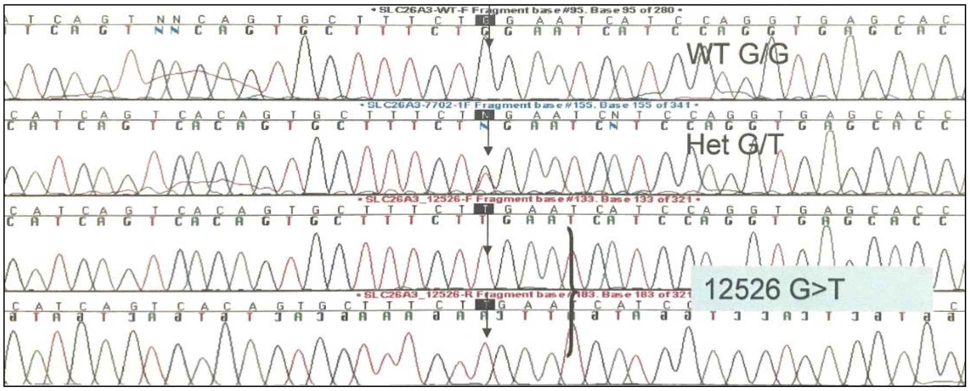

G5

PCCA

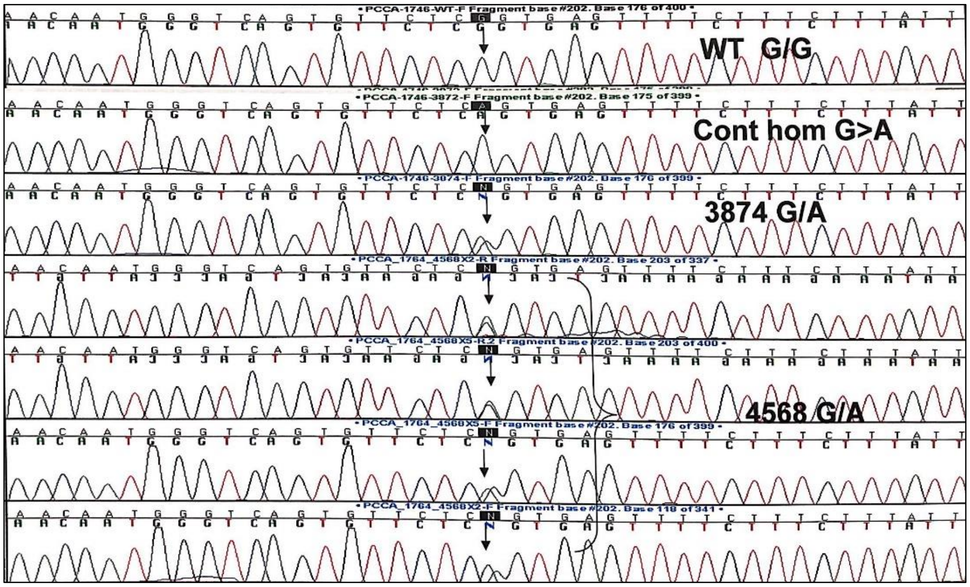

G5

FKBP10

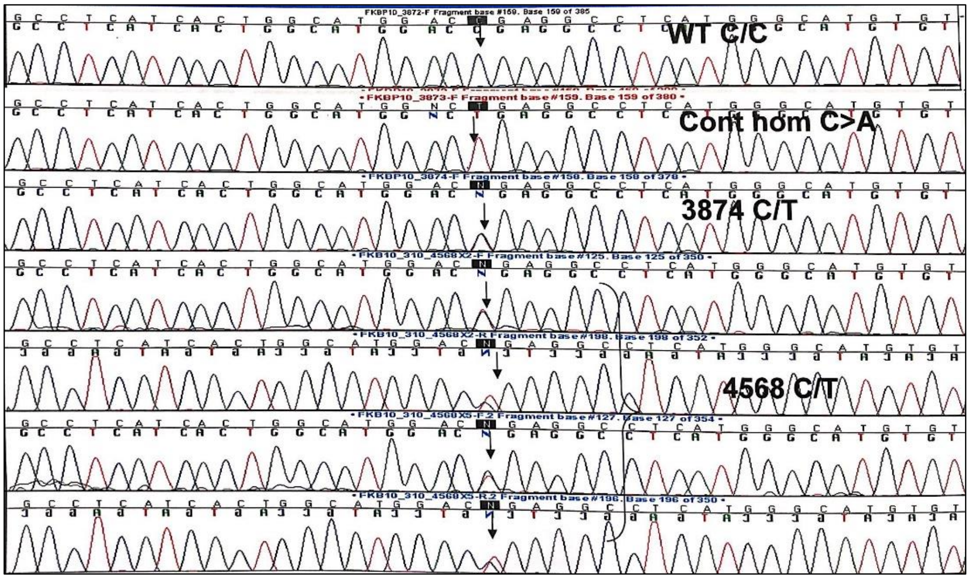

Supplemental Figure S3. Mutation validation Sanger results.

The mutations families E1 and G5 were confirmed using Sanger. In E2, the first row demonstrates a control that is homozygous to the reference allele (G). The second row shows the father, who is a carrier of the alternate allele (T). The third and fourth rows show the results of the fetus, once using a forward primer and once using a reverse primer. The fetus is homozygous to the alternate allele. In both cases in G5, rows 1-2 represent the control, row 3 represents the mother, and rows 4-7 represent the fetus.
