# Supplementary material for: Bayesian-based noninvasive prenatal diagnosis of single-gene disorders
Source: Genome Res. 2019 Mar;29(3):428–38. doi: 10.1101/gr.235796.118 (PMC6396420; doi:10.1101/gr.235796.118)
Supplement: Supplemental Material [file supp_gr.235796.118_Supplemental_Fig_S4.pdf]

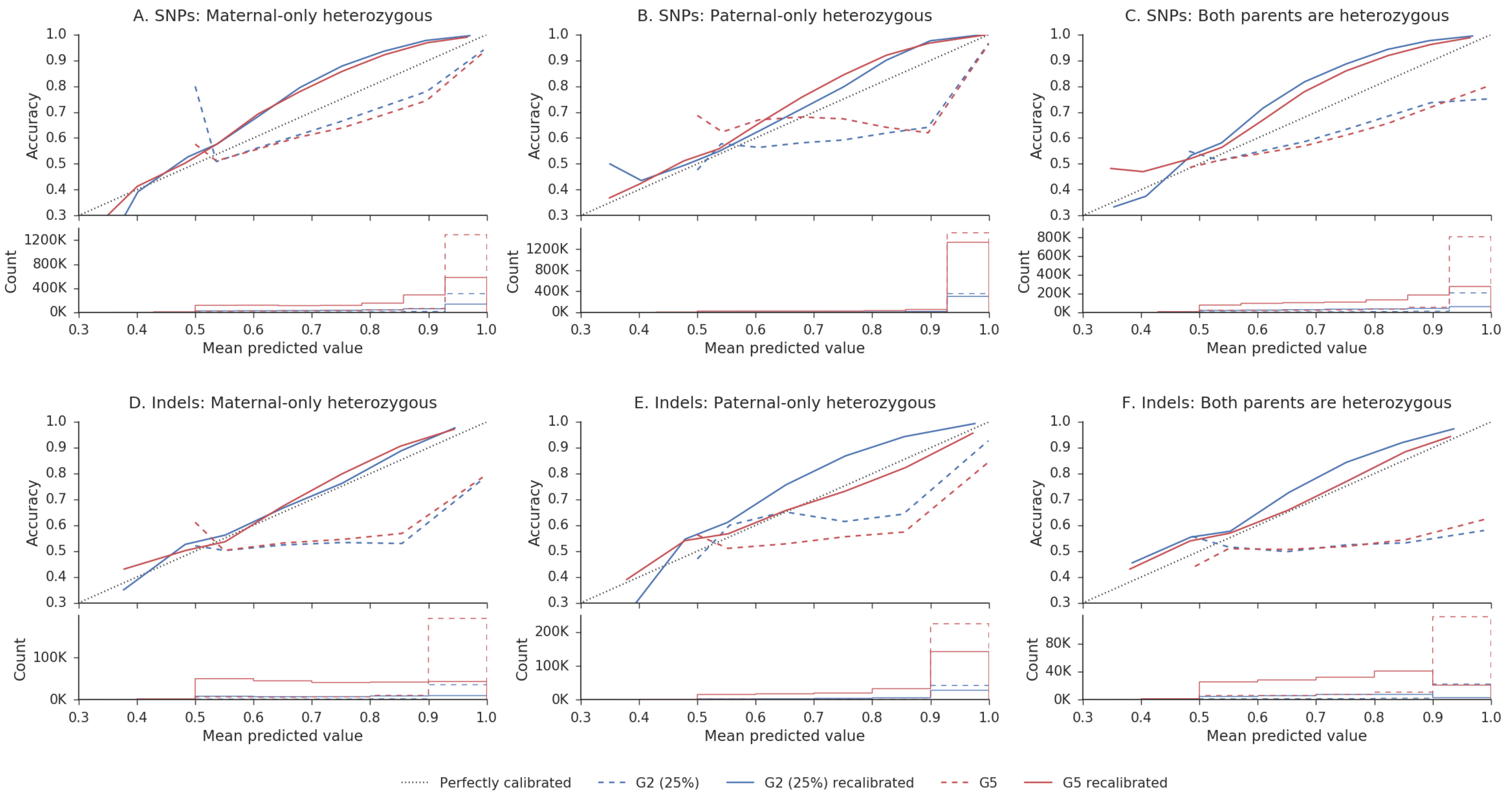

Supplemental Figure S4. Calibration of the model's accuracy.

Shown here is the accuracy as a function of the prediction probability, before and after variant recalibration. The "perfectly calibrated" line represents a situation in which the probability assigned to a prediction perfectly describes the probability of being correct (in terms of accuracy). Presented in the lower pane of each figure is the marginal distribution of loci in each bin.
