# Supplementary material for: Bayesian-based noninvasive prenatal diagnosis of single-gene disorders
Source: Genome Res. 2019 Mar;29(3):428–38. doi: 10.1101/gr.235796.118 (PMC6396420; doi:10.1101/gr.235796.118)
Supplement: Supplemental Material [file supp_gr.235796.118_Supplemental_Fig_S5.pdf]

Mother	Father	cfDNA	Illustration
A/A	G/G		1 GGGGGGGGGGGAAAAAAAAAAAAAAAAAAAAAAAAAAAAAAAAAAAAAA
A/A	A/G	G detected	2 GGGG AAAAAAAAAAAAAAAAAAAAAAAAAAAAAAAAAAAAAA
			3 GGGG AAAAAAAAAAAAAAAAAAAAAAAAAAAAAAAAAAAAAA
			4 GGGGAAAAAAAAAAAAAAAAAAAAAAAAAAAAAAAAAAAAAAAAAAAAA
A/A	A/G	G not detected	1 AGGAAGAAAGAAGAAAGAAGGGAAAAGAAAAAAAAGAAGGAAGAAGAGG
A/A	A/A		2 AGGAAGAAAGAAGAAAGAAGGGAAAAGAAAAAAAAGAAGGAAGAAGAGG
A/G	all	all	

Supplemental Figure S5. Fetal fraction reduction method.

Red and blue indicate the probability of a read being maternal or fetal, respectively, based on its length, and on the presented allele, parental genotypes at the given loci, and fetal DNA fraction within the cfDNA. In the first two parental genotype combinations presented in the table: (1) on the left side of the illustration, a fetal-specific allele G can be identified, and on the right, the shared allele A is shown, while the gradient demonstrates the overlap between the two length distributions; (2) $N = 1 - d/f$ reads presenting the fetal-specific allele are discarded (d – desired fetal fraction, f – observed fraction); (3) N reads presenting the shared allele are sampled based on fetal length distribution, and are assigned with new length values from the maternal distribution; (4) N reads presenting the shared allele are added and are assigned with length from the maternal distribution.

In all the other scenarios: (1) no fetal-specific allele can be identified; (2) $N = 1 - d/f$ of all the reads are sampled based on the fetal length distribution, and are assigned with lengths from the maternal distribution.
