# Supplementary material for: Bayesian-based noninvasive prenatal diagnosis of single-gene disorders
Source: Genome Res. 2019 Mar;29(3):428–38. doi: 10.1101/gr.235796.118 (PMC6396420; doi:10.1101/gr.235796.118)
Supplement: Supplemental Material [file supp_gr.235796.118_Supplemental_Code.pdf]

```
# BSD 3-Clause License
# Copyright (c) 2019 Noam Shomron, Tom Rabinowitz
# All rights reserved.
# Redistribution and use in source and binary forms, with or without modification, are permitted provided that the following conditions are met:
# * Redistributions of source code must retain the above copyright notice, this list of conditions and the following disclaimer.
# * Redistributions in binary form must reproduce the above copyright notice, this list of conditions and the following disclaimer in the documentation and/or
#   other materials provided with the distribution.
# * Neither the name of the copyright holder nor the names of its contributors may be used to endorse or promote products derived from this software without
#   specific prior written permission.
# THIS SOFTWARE IS PROVIDED BY THE COPYRIGHT HOLDERS AND CONTRIBUTORS "AS IS" AND ANY EXPRESS OR IMPLIED WARRANTIES, INCLUDING, BUT NOT LIMITED TO, THE IMPLIED
# WARRANTIES OF MERCHANTABILITY AND FITNESS FOR A PARTICULAR PURPOSE ARE DISCLAIMED. IN NO EVENT SHALL THE COPYRIGHT HOLDER OR CONTRIBUTORS BE LIABLE FOR ANY
# DIRECT, INDIRECT, INCIDENTAL, SPECIAL, EXEMPLARY, OR CONSEQUENTIAL DAMAGES (INCLUDING, BUT NOT LIMITED TO, PROCUREMENT OF SUBSTITUTE GOODS OR SERVICES; LOSS OF
# USE, DATA, OR PROFITS; OR BUSINESS INTERRUPTION) HOWEVER CAUSED AND ON ANY THEORY OF LIABILITY, WHETHER IN CONTRACT, STRICT LIABILITY, OR TORT (INCLUDING
# NEGLIGENCE OR OTHERWISE) ARISING IN ANY WAY OUT OF THE USE OF THIS SOFTWARE, EVEN IF ADVISED OF THE POSSIBILITY OF SUCH DAMAGE.

# --------- import modules ------------
# external
import re
import os
import sys
import subprocess
import requests
import vcf, vcf.utils
import numpy as np
import pandas as pd
from collections import OrderedDict
import pickle
import time
# project's
import parse_gt
from stderr import *
import vcfuid
import position
import vcf_out
import preprocessing
from arguments import args
from db import Variants

# connect to the database that was created during the first analysis of the cfDNA sample
if args.region and not args.db:
	printerr('a region was specified, setting default db as tmp_folder/chrX:START-END.db. \
		you can change that by specifying --tmp_dir or --db')
	args.db = os.path.join(args.tmp_dir, args.region + '.db')
vardb = Variants(args.db, probe=False)

# pre-processing
# calculate the total fetal fraction and a table of fetal-fraction per fragment size
err_rate, total_fetal_fraction, fetal_fractions_df = preprocessing.run_full_preprocessing(	args.db,
											args.fetal_sample_name,
											args.fetal_fraction,
											args.calculate_empirical_ff_dist,
											args.cores,
											window = args.window,
											max_len = 500,
											plot = args.plot_lengths,
											qnames = args.qnames,
											region = args.region)
# create vcf files iterators
cfdna_reader = vcf.Reader(filename = args.cfdna_vcf)
parents_reader = vcf.Reader(filename = args.parents_vcf)

# print header of output vcf file
input_command = ' '.join(sys.argv)
```

```
vcf_out.make_header(   cfdna_reader,
                       parents_reader,
                       input_command,
                       total_fetal_fraction,
                       args.fetal_sample_name,
                       vcf_out.reserved_formats,
                       output_path = args.vcf_output)

# fetch region, if a region was specified
if args.region:
        region_split = re.split(':|-', args.region)
        chrom = region_split[0]
        start = int(region_split[1]) if len(region_split) > 1 else None
        end = int(region_split[2]) if len(region_split) > 2 else None
        try:
                cfdna_reader = cfdna_reader.fetch(chrom, start, end)
                parents_reader = parents_reader.fetch(chrom, start, end)
        except ValueError as e:
                errmessage = e.args[0]
                if 'could not create iterator for region' in errmessage:
                        sys.exit('warning! ' + errmessage + ', probably the input file does not contain any variants in the region.')

# get sample of cfdna from its vcf file, and of the parents from the input arguments
cfdna_id = cfdna_reader.samples[0]
mother_id, father_id = vardb.get_samples()

# processing positions
# iterate on both vcf files and return a tuple for each position, that contains its record from each vcf file.
# if there is no vcf record for a certain position in one of the files, a None will appear in the tuple instead.
co_reader = vcf.utils.walk_together(cfdna_reader, parents_reader)
for tup in co_reader:
        # reset prediction and QUAL
        prediction = qual = None
        rec_info = []

        cfdna_rec, parents_rec = tup

        printverbose('parents_rec: ', parents_rec)
        printverbose('cfdna_rec: ', cfdna_rec)

        if not parents_rec:
                vcf_out.unsupported_position(cfdna_rec, out_path = args.vcf_output)
        else: # if parental and cfdna record

                # fetch parental genotypes
                maternal_gt = parse_gt.str_to_int(parents_rec.genotype(mother_id).data.GT)
                paternal_gt = parse_gt.str_to_int(parents_rec.genotype(father_id).data.GT)
                printverbose(maternal_gt, paternal_gt)


                if not cfdna_rec:
                        cfdna_rec = parents_rec
                        qual = 0
                        cfdna_geno_sample_dic = '.'
                elif maternal_gt not in (0,1,2):
                        qual = 0
                        cfdna_geno_sample_dic = '.'
                else:
                        # for now, only positions where the mother is 0/0, 0/1 or 1/1 are supported
                        if maternal_gt in (0,1,2):

                                # calculate priors for the position
                                priors = position.calculate_priors(maternal_gt, paternal_gt)

                                # calculate likelihoods for the position
                                likelihoods = position.calculate_likelihoods(       cfdna_rec,
```

```
                                                                            maternal_gt,
                                                                            total_fetal_fraction,
                                                                            fetal_fractions_df,
                                                                            err_rate,
                                                                            vardb,
                                                                            args.model)

                # calculate posteriors for the position
                posteriors, prediction, qual = position.calculate_posteriors(priors, likelihoods)


                ## process the output entry

                # create normalized likelihoods for the output vcf
                normalized_likelihoods = position.likelihoods_to_phred_scale(likelihoods)

                # fetal information for the sample and FORMAT fields
                cfdna_geno_sample_dic = vcf_out.rec_sample_to_string(cfdna_rec, cfdna_id)
                if cfdna_geno_sample_dic != '.':
                        cfdna_geno_sample_dic['GT'] = parse_gt.int_to_str(prediction)
                        cfdna_geno_sample_dic['GL'] = (','.join(str(round(p,2)) for p in list(normalized_likelihoods)))
                        cfdna_geno_sample_dic['PG'] = (','.join(str(round(p,5)) for p in list(priors)))
                        cfdna_geno_sample_dic['PP'] = (','.join(str(round(p,5)) for p in list(posteriors)))

                rec_info.append(vcf_out.info_to_string(cfdna_rec.INFO))

        # for each parent, for all the data in its sample, create an instance that will be printed in the output INFO
        rec_info.append(vcf_out.parents_gt_to_info(mother_id, father_id, parents_rec))

        parental_info_for_info = vcf_out.info_to_string(parents_rec.INFO)
        parental_info_for_info = 'P' + parental_info_for_info.replace(';',';P')
        rec_info.append(parental_info_for_info)

        rec_info = ';'.join(rec_info)

        rec_info = rec_info.replace('None', '.')

        # write var out (to file passed with -v or to output)
        vcf_out.print_var(cfdna_rec, qual, rec_info, cfdna_geno_sample_dic, out_path = args.vcf_output)
```

Arguments.py

```
import argparse
import os

tmp_dir = os.path.join(os.getcwd(), 'tmp_hb')

parser = argparse.ArgumentParser()

parser.add_argument("-f", "--fetal_sample_name", default = 'FETUS', help = 'fetal sample name to write in the outputvcf')
parser.add_argument("-parents_vcf", "--parents_vcf", help = 'The maternal plasma cfDNA VCF file')
parser.add_argument("-cfdna_vcf", "--cfdna_vcf", help = 'The maternal plasma cfDNA VCF file')
parser.add_argument("-t", "--tmp_dir", default = tmp_dir, help = 'Directory for temporary files')
parser.add_argument("-o", "--vcf_output", default = False, help = 'path for vcf output')
parser.add_argument("-r", "--region", default = False, help = "run on a specific region as explained in pyvcf documentation")
parser.add_argument("-v", "--verbosity", action = 'store_true', help = "Prints more detailed debugging information")
parser.add_argument("-w", "--window", default = 3, help = "Window size for lengths")
parser.add_argument("-%", "--fetal_fraction", default = False, help = "specify a known fetal fraction to pass hoobari's calculation of it")
parser.add_argument("-e", "--calculate_empirical_ff_dist", default = True, help = "whether to calculate empricial distribution \
                                                                                  of fetal fractions per fragment length")
parser.add_argument("-l", "--plot_lengths", default = False, action = 'store_true', help = "Creates a plot of the length distributions")
parser.add_argument("-q", "--qnames", default = False, action = 'store_true', help = "Creates lists of fetal and shared qnames")
parser.add_argument("-d", "--db", default = os.path.join(tmp_dir, 'hoobari.db'), help = 'db path')
parser.add_argument("-@", "--cores", default = 1, help = 'number of cores to run pre-processing when run split')
parser.add_argument("-model", "--model", default = 'lengths', help = '     model for likelihoods calculation. possible values: "simple" \
```

```
                                                                    (Bayesian model based only on fetal fraction and parental genotypes), \
                                                                    "lengths" (use different fetal fraction per fragment length), \
                                                                    "origin" (use fragments that are very likely to be fetal, \
                                                                    based on other SNPs on these fragments)')
args = parser.parse_args()
```

db.py

```
import sqlite3
import os
import pandas as pd

class Variants(object):
    def __init__(self, dbpath = './hoobari.db', probe=True):

        # create directory
        os.makedirs(os.path.dirname(dbpath), exist_ok=True)

        # Connect to DB
        self.con = sqlite3.connect(dbpath, isolation_level = None)

        # Check table's existance
        if probe:
            # Drop existing database if needed
            res_list = [i[0:2] for i in self.con.execute("SELECT type, name FROM sqlite_master WHERE type in ('table','view')").fetchall()]
            for res in res_list:
                self.con.execute('DROP ' + res[0] + ' IF EXISTS ' + res[1])

            self.con.execute(   '''CREATE TABLE qnames(
                                qname varchar(50) PRIMARY KEY,
                                is_fetal tinyint(1) DEFAULT NULL
                                )''')
            # self.con.execute('CREATE INDEX idx_length_fetal ON qnames (length, for_ff)')
            self.con.execute(   '''CREATE TABLE variants(
                                qname varchar(50) NOT NULL,
                                chromosome char(2) DEFAULT NULL,
                                pos int(10) NOT NULL,
                                genotype varchar(50) DEFAULT NULL,
                                var_type tinyint(1) DEFAULT NULL,
                                length int(10) DEFAULT NULL,
                                for_ff tinyint(1) DEFAULT NULL,
                                is_fetal tinyint(1) DEFAULT NULL,
                                FOREIGN KEY(qname) REFERENCES qnames(qname)
                                )''')
            self.con.execute('CREATE INDEX idx_chrom_pos ON variants (chromosome, pos)')
            self.con.execute(   '''CREATE TABLE samples(
                                mother char(20) DEFAULT NULL,
                                father char(20) DEFAULT NULL)''')

    # Insert variants to table
    def insertVariant(self, chromosome, position, info_list):

        query = '''
            INSERT INTO `variants`
            (qname,
            chromosome,
            pos,
            genotype,
            var_type,
            length,
            for_ff,
            is_fetal)
            VALUES
            '''
```

```
    for line in info_list:
        query += '("{0}","{1}",{2},"{3}",{4},{5},{6},{7}),'.format(line[2],
                chromosome, position, line[0], line[4], line[1], line[5], line[3])

    query = query[:-1]
    self.con.execute(query)
    self.con.commit()

# Create qnames table for origin model
def createQnamesTable(self):
    self.con.execute('''INSERT INTO qnames
                                SELECT qname, max(is_fetal)
                                from variants
                                group by qname''')

# Create views for both shared and fetal lengths
def lengthDists(self):
    self.con.execute('''
    CREATE VIEW fetal_lengths AS SELECT length, qname FROM variants WHERE for_ff=1 GROUP BY qname
    ''')
    self.con.execute("""
                    CREATE TABLE fetal_length_counts
                    AS SELECT length, COUNT(length)
                    FROM fetal_lengths
                    GROUP BY length
                    """)
    self.con.execute('''
    CREATE VIEW shared_lengths AS SELECT length, qname FROM variants WHERE for_ff=2 GROUP BY qname
    ''')
    self.con.execute("""
                    CREATE TABLE shared_length_counts
                    AS SELECT length, COUNT(length)
                    FROM shared_lengths
                    GROUP BY length
                    """)

# Get fetal lengths
def getFetalLengths(self):
    return pd.read_sql_query("select * from fetal_length_counts", self.con).set_index('length')

# Get shared lengths
def getSharedLengths(self):
    return pd.read_sql_query("select * from shared_length_counts", self.con).set_index('length')

# Gets fetal and shared qnames
def getFetalSharedQnames(self):
    fetal_qnames = set([i[0] for i in self.con.execute("SELECT DISTINCT(qname) FROM fetal_lengths")])
    shared_qnames = set([i[0] for i in self.con.execute("SELECT DISTINCT(qname) FROM shared_lengths")])
    return fetal_qnames, shared_qnames

# Gets all variants in specified chromosomal position
def getPositionVariants(self, chromosome, position, model):
    if model == 'origin':
        return self.con.execute("""
                            SELECT v.genotype, v.length, q.is_fetal
                            FROM variants v, qnames q
                            WHERE v.chromosome=:chr AND v.pos=:pos AND q.qname=v.qname
                """,{"chr":chromosome, "pos":position})
    else:
        return self.con.execute("""
                            SELECT genotype, length, is_fetal
                            FROM variants
                            WHERE chromosome=:chr AND pos=:pos
                """,{"chr":chromosome, "pos":position})
```

```
    def create_samples_table(self, parental_samples):
        mother, father = parental_samples
        self.con.execute('INSERT INTO `samples` (mother, father) VALUES (:m, :f)', {'m': str(mother), 'f': str(father)})

    def get_samples(self):
        return self.con.execute('SELECT * FROM samples').fetchall()[0]
```

parse_gt.py

```
def str_to_int(str_gt):
        if str_gt in ('0/0', '0/1', '1/1'):
                gt = int(str_gt[0]) + int(str_gt[2])
        elif str_gt == '.':
                gt = None
        else:
                gt = 'unsupported'

        return gt

def int_to_str(gt):
        if gt == 0:
                string = '0/0'
        elif gt == 1:
                string = '0/1'
        elif gt == 2:
                string = '1/1'
        else:
                string = '.'
        return string
```

position.py

```
# --------- import modules ------------
# external
import os
import requests
import numpy as np
import time
import argparse
from multiprocessing import Pool, cpu_count
import ctypes
from decimal import *
# project's
import parse_gt
from stderr import *
import vcfuid

# --------- global -------------
de_novo = 1.2e-8
valid_gts = (0,1,2)
default_decimal_prec = getcontext().prec

# --------- functions ----------
def calculate_priors(maternal_gt, paternal_gt):

        '''
        Calculate prior probabilities for each of the 3 possible fetal genotypes (1/1, 0/1 and 0/0), given the parents genotypes.
        Mother: Calculations below assume maternal genotype = '0/1', since these are the positions of interest. Therefore the
        probability to inherit the alternate allele from her is 0.5.
        Father: If the father is 1/1 (2) the probability to inherit the alternate allele is 1. if he's 0/1 (1) it's 0.5,
        and if he's 0/0 (0) it's 0. therefore it's always the (sum_of_alternate_alleles/2), which is marked is f.
        P(fetus = 1/1) = p_inherit_alt_from_mother * p_inherit_alt_from_father = 0.5 * f
        P(fetus = 0/1) = (p_inherit_alt_from_mother * p_inherit_ref_from_father) + (p_inherit_ref_from_mother * p_inherit_alt_from_father) =
        0.5 * (1 - f) + 0.5 * f = 0.5 * (1 - f + f) = 0.5
        P(fetus = 0/0) = p_inherit_ref_from_mother * p_inherit_ref_from_father = 0.5 * (1 - f)
        ==>
```

```
        [0.5f, 0.5, 0.5(1-f)]
        '''

        if (maternal_gt in valid_gts) and (paternal_gt in valid_gts):
                p_maternal_alt = maternal_gt / 2
                p_paternal_alt = paternal_gt / 2
                priors = np.array([    (1-p_maternal_alt)*(1-p_paternal_alt),
                                       p_maternal_alt*(1-p_paternal_alt) + (1-p_maternal_alt)*p_paternal_alt,
                                       p_maternal_alt*p_paternal_alt])
        else:
                priors = np.array([1/3, 1/3, 1/3])

        printverbose('priors:', priors)

        return priors

def calculate_fragment_i(frag_genotype, maternal_gt, ref, alt, f, err_rate):
        '''
        probabilities of each fragment i to show the reference allele, given one of the 3 possible fetal genotypes.
        P(frag_i | fetal_genotype) = P(frag_i | frag from fetus)P(frag from fetus | fetal_genotype) + P(frag_i | frag from mother)P(frag from mother |
fetal_genotype)
        the first expression is the fetal quantity of the allele, and the second is the maternal quantity of the allele.
        example: fetal fraction (ff is 0.1, fetus is aa, mother Aa. Therefore the fetus donates 0.1 fragments with genotype a, and half of the maternal fragments,
        which is (1-0.1)/2 = 0.45, also donate genotype a => ff + (1-ff)*0.5 = 2ff*0.5 + (1-ff)*0.5 = 0.5(1 - ff + 2ff) = 0.5(1 + ff)
        if fetus is aA - 0.5*ff + 0.5*(1-ff) = 0.5(ff+1-ff) = 0.5
        if fetus is AA - 0*ff + 0.5(1-ff) = 0.5(1-ff)
        '''

        # 0 would cause -inf after log, and the sum would also be -inf

        # fetal genotypes: 0,1,2
        # print(frag_genotype)
        if frag_genotype == alt:
                p_maternal_alt = max(err_rate, maternal_gt / 2)
                frag_i_likelihoods = np.array([       0*f + p_maternal_alt*(1-f), # fetal is 0/0
                                               0.5*f + p_maternal_alt*(1-f), # fetal is 0/1
                                               1*f + p_maternal_alt*(1-f)]) # fetal is 1/1

        elif frag_genotype == ref:
                p_maternal_ref = max(err_rate, 1 - (maternal_gt / 2))
                frag_i_likelihoods = np.array([       1*f + p_maternal_ref*(1-f), # fetal is 0/0
                                               0.5*f + p_maternal_ref*(1-f), # fetal is 0/1
                                               0*f + p_maternal_ref*(1-f)]) # fetal is 1/1

        else:
                frag_i_likelihoods = np.array([1, 1, 1])

        return frag_i_likelihoods

def calculate_likelihoods(
        rec,
        maternal_gt,
        total_fetal_fraction,
        fetal_fractions_df,
        err_rate,
        vardb,
        model,
        **kwargs):

        '''
        calculate likelihoods for each of the 3 possible fetal genotype, based on the prbability that
        fragment_i at the position, will show a certain allele (ref or alt), given other factors of
        the model, such as the maternal genotype, fragment length and the fetal genotype (which is unknown,
        so we check for all possibilities - 1/1, 0/1 and 0/0)
        '''
```

```
	chrom, pos, ref, alt = rec.CHROM.replace('chr', ''), str(rec.POS), rec.REF, str(rec.ALT[0])

	variant_len = len(ref) - len(alt)

	printverbose(chrom, pos)
	pos_data = vardb.getPositionVariants(chrom, pos, model).fetchall()
	printverbose('position_data:')
	printverbose(pos_data)

	first = 1
	if (len(pos_data) > 0) and (maternal_gt in valid_gts):

		for frag_genotype, frag_length, frag_is_fetal in pos_data:

			frag_length = max(int(frag_length) - variant_len, 0)

			# get fetal fraction
			if (model == 'origin') and (frag_is_fetal == 1):
				printverbose('fragment was marked as fetal by another variant')
				ff = 1 - err_rate
			elif (model in ('lengths', 'origin')) and (frag_length in fetal_fractions_df.index.values):
				ff = fetal_fractions_df[frag_length]
			else:
				ff = total_fetal_fraction

			frag_i_likelihoods = calculate_fragment_i(frag_genotype, maternal_gt, ref, alt, ff, err_rate)

			#frag_i_likelihoods[frag_i_likelihoods == 0] = 0.003
			frag_i_likelihoods = np.log(frag_i_likelihoods, dtype = np.float64)
			if first:
				sum_log_fragments_likelihoods_df = frag_i_likelihoods
				first = 0
			sum_log_fragments_likelihoods_df = np.add(sum_log_fragments_likelihoods_df, frag_i_likelihoods)
			printverbose(ff, frag_i_likelihoods, sum_log_fragments_likelihoods_df, sep = '\t')

	else:
		sum_log_fragments_likelihoods_df = np.log([1, 1, 1], dtype = np.float64)
	printverbose(sum_log_fragments_likelihoods_df)

	return sum_log_fragments_likelihoods_df

def simple_qual_calculation(posteriors):

	if posteriors[0] == 0:
		return '1e+06'
	else:
		qual = -10 * np.log10(posteriors[0])

		if qual == 0:
			return int(0)
		elif qual < 1:
			return '{:0.3e}'.format(qual)
		else:
			return round(qual,2)

def likelihoods_to_phred_scale(likelihoods):

	log10_likelihoods = likelihoods / np.log(10)
	normalized_log10_likelihoods = log10_likelihoods - np.max(log10_likelihoods)
	phred_scaled_normalized_likelihoods = -10 * (normalized_log10_likelihoods)
	phred_scaled_normalized_likelihoods[phred_scaled_normalized_likelihoods == -0.0] = 0
	printverbose('normalized_likelihoods', phred_scaled_normalized_likelihoods)

	return phred_scaled_normalized_likelihoods

def calculate_posteriors(var_priors, var_likelihoods):
```

```
	printverbose(var_priors, var_likelihoods, sep = '\n')
	# sum priors and likelihoods
	# if there are no priors (for instance if parental genotypes at positions are missing),
	# take only likelihoods

	var_priors = np.log(var_priors)
	printverbose('log(priors):', var_priors)

	joint_probabilities = np.add(var_priors, var_likelihoods, dtype = np.float64)
	prediction = joint_probabilities.argmax()

	joint_probabilities_normalized = joint_probabilities - np.min(joint_probabilities[np.isfinite(joint_probabilities)])
	exp_joint_probabilities = np.exp(joint_probabilities_normalized)
	printverbose('joint_probabilities_normalized:', joint_probabilities_normalized)
	posteriors = np.asarray((exp_joint_probabilities / np.sum(exp_joint_probabilities)), dtype = np.float64)
	printverbose('posteriors:', posteriors)
	if np.inf in exp_joint_probabilities:
		getcontext().prec = default_decimal_prec
		start_time = time.time()
		while 1 or np.nan in posteriors:
			if getcontext().prec <= 2**11:
				printverbose('precision:', str(getcontext().prec))
				exp_joint_probabilities = np.array([Decimal(i).exp() for i in joint_probabilities_normalized])
				posteriors = np.array(exp_joint_probabilities / np.sum(exp_joint_probabilities))
				printverbose('posteriors:', posteriors)
				getcontext().prec *= 2
			else:
				break
		printverbose(time.time() - start_time)

	qual = simple_qual_calculation(posteriors)

	printverbose('Posteriors:', posteriors)
	printverbose('Predicted genotype:', prediction)
	printverbose('QUAL:', qual)

	return (posteriors, prediction, qual)
```

preprocessing.py

```
# --------- import modules ------------
# external
import os, sys
import sqlite3
import vcf
from collections import Counter
from numpy import repeat as nprepeat
import pandas as pd
import numpy as np
import matplotlib
matplotlib.use('Agg') # to allow saving a figure even though display is invalid
import matplotlib.pyplot as plt
import seaborn as sns
from multiprocessing import Pool, cpu_count
from functools import partial
import re

# project's
import parse_gt
from stderr import *
import vcfuid
import db


# --------- functions ----------
```

```
def get_fetal_and_shared_lengths(db_path, qnames = False):
	'''
	input - path to the database from hoobari's patch
	output - a tuple with two dictionaries, one contains the counts of fetal fragments at different lengths,
	and the other is similar, but for fragments which aren't necessarily fetal ("shared")
	'''

	con = db.Variants(db_path, probe=False)
	fetal_lengths = con.getFetalLengths()
	shared_lengths = con.getSharedLengths()

	if qnames:
		fetal_qnames, shared_qnames = con.getFetalSharedQnames()
		return (shared_lengths, fetal_lengths, shared_qnames, fetal_qnames)
	else:
		return (shared_lengths, fetal_lengths)


def create_length_distributions(db_path, cores, qnames = False, region = False):
	'''
	input: db_path - path to the database from hoobari's patch; cores - number of cores to use for
	multiprocessing; db_prefix - if hoobari's patch was ran for many different regions, it creates
	many DB's with the same prefix. since all those databases are required in this step, the prefix
	is also required.
	output: a tuple with two pandas dataframes that contain fragment length distributions which were
	calculated using *all* the databases
	'''

	pool = Pool(int(cores))

	# if db_prefix was given as an argument, work for all databases with that prefix.
	db_path = os.path.abspath(db_path)
	db_dir = os.path.dirname(db_path)
	db_files = [os.path.join(db_dir, file) for file in os.listdir(db_dir) if file.endswith('.db')]

	# run the function get_fetal_and_shared_lengths for each path in db_files
	get_qnames_and_alleles_with_args = partial(get_fetal_and_shared_lengths, qnames = qnames)

	# create two lists, one with all the shared fragments results, and one for the fetal fragments results

	con = db.Variants(db_files[0], probe=False)
	try:
		shared_lengths = con.getSharedLengths()
		fetal_lengths = con.getFetalLengths()
	except:
		printerr(db_files[0])

	if qnames:
		fetal_qnames_set, shared_qnames_set = con.getFetalSharedQnames()

	for tup in pool.imap_unordered(get_qnames_and_alleles_with_args, db_files[1:]):
		shared_lengths = shared_lengths.add(tup[0], fill_value=0)
		fetal_lengths = fetal_lengths.add(tup[1], fill_value=0)
		if qnames:
			shared_qnames_set.update(tup[2])
			fetal_qnames_set.update(tup[3])
	if qnames:
		with open('shared_qnames_list.txt', 'w') as f:
			for q in shared_qnames_set:
				print(q, file = f)
		with open('fetal_qnames_list.txt', 'w') as f:
			for q in fetal_qnames_set:
				print(q, file = f)

	return(shared_lengths, fetal_lengths)
```

```
def estimate_length_distribution(total_fetal_fraction):
        normalized_fetal_fractions_dist = pd.Series([1.195695703013115, 1.195695703013115, 1.195695703013115, 1.195695703013115, 2.1292505317701584,
2.1292505317701584, 2.1292505317701584, 2.266846530994138, 2.266846530994138, 2.266846530994138, 1.500350157313987, 1.500350157313987, 1.500350157313987,
1.4017112196359047, 1.4017112196359047, 1.4017112196359047, 2.1773657468759486, 2.1773657468759486, 2.1773657468759486, 1.2377350652159864, 1.2377350652159864,
1.2377350652159864, 1.5465401566595522, 1.5465401566595522, 1.5465401566595522, 2.4365123449703865, 2.4365123449703865, 2.4365123449703865, 1.4853524240997755,
1.4853524240997755, 1.4853524240997755, 1.3845453630979483, 1.3845453630979483, 1.3845453630979483, 1.502413694347957, 1.502413694347957, 1.502413694347957,
1.4704651566050584, 1.4704651566050584, 1.4704651566050584, 1.3658814263443844, 1.3658814263443844, 1.3658814263443844, 1.3763656011350447, 1.3763656011350447,
1.3763656011350447, 1.4051395839047558, 1.4051395839047558, 1.4051395839047558, 1.418233691511955, 1.418233691511955, 1.418233691511955, 1.3090329283666646,
1.3090329283666646, 1.3090329283666646, 1.431968780624545, 1.431968780624545, 1.431968780624545, 1.5523974278336532, 1.5523974278336532, 1.5523974278336532,
1.5055667413383695, 1.5055667413383695, 1.5055667413383695, 1.5402599292330517, 1.5402599292330517, 1.5402599292330517, 1.6017058738173935, 1.6017058738173935,
1.6017058738173935, 1.8423934249381364, 1.8423934249381364, 1.8423934249381364, 1.5959188109726787, 1.5959188109726787, 1.5959188109726787, 1.6819447781494345,
1.6819447781494345, 1.6819447781494345, 1.838203977414241, 1.838203977414241, 1.838203977414241, 1.7224392310333196, 1.7224392310333196, 1.7224392310333196,
1.7289746387048759, 1.7289746387048759, 1.7289746387048759, 1.9095170280669445, 1.9095170280669445, 1.9095170280669445, 2.072064953768226, 2.072064953768226,
2.072064953768226, 2.031656551788986, 2.031656551788986, 2.031656551788986, 2.0585066571357045, 2.0585066571357045, 2.0585066571357045, 2.3809272197788394,
2.3809272197788394, 2.3809272197788394, 2.1077576575995667, 2.1077576575995667, 2.1077576575995667, 2.191545927606252, 2.191545927606252, 2.191545927606252,
2.2134804031902116, 2.2134804031902116, 2.2134804031902116, 2.265610562618742, 2.265610562618742, 2.265610562618742, 2.2168216834122103, 2.2168216834122103,
2.2168216834122103, 2.218832271923642, 2.218832271923642, 2.218832271923642, 2.2368565308945136, 2.2368565308945136, 2.2368565308945136, 2.25614804726495,
2.25614804726495, 2.25614804726495, 2.254777147721188, 2.254777147721188, 2.254777147721188, 2.174109728454312, 2.174109728454312, 2.174109728454312,
2.0921539080118357, 2.0921539080118357, 2.0921539080118357, 1.9909559873678913, 1.9909559873678913, 1.9909559873678913, 1.8062678281374691, 1.8062678281374691,
1.8062678281374691, 1.7368734604292917, 1.7368734604292917, 1.7368734604292917, 1.6218278923731848, 1.6218278923731848, 1.6218278923731848, 1.4322992178660081,
1.4322992178660081, 1.4322992178660081, 1.3352647823898771, 1.3352647823898771, 1.3352647823898771, 1.2686590417033243, 1.2686590417033243, 1.2686590417033243,
1.0927236004122058, 1.0927236004122058, 1.0927236004122058, 0.9803002372280191, 0.9803002372280191, 0.9803002372280191, 0.8427104331050591, 0.8427104331050591,
0.8427104331050591, 0.7381302643241816, 0.7381302643241816, 0.7381302643241816, 0.7075162035381661, 0.7075162035381661, 0.7075162035381661, 0.676098160742928,
0.676098160742928, 0.676098160742928, 0.6278752302805902, 0.6278752302805902, 0.6278752302805902, 0.6231931763990364, 0.6231931763990364, 0.6231931763990364,
0.63257035977172, 0.63257035977172, 0.63257035977172, 0.6139473479557893, 0.6139473479557893, 0.6139473479557893, 0.5857105932383947, 0.5857105932383947,
0.5857105932383947, 0.5881352460327547, 0.5881352460327547, 0.5881352460327547, 0.594082727260782, 0.594082727260782, 0.594082727260782, 0.586817647502322,
0.586817647502322, 0.586817647502322, 0.5698097918562272, 0.5698097918562272, 0.5698097918562272, 0.5806322325263193, 0.5806322325263193, 0.5806322325263193,
0.5904635937592888, 0.5904635937592888, 0.5904635937592888, 0.5840741907923023, 0.5840741907923023, 0.5840741907923023, 0.5840255259523812, 0.5840255259523812,
0.5840255259523812, 0.5922311565838844, 0.5922311565838844, 0.5922311565838844, 0.6064678225478712, 0.6064678225478712, 0.6064678225478712, 0.6136005641432725,
0.6136005641432725, 0.6136005641432725, 0.6244629891099108, 0.6244629891099108, 0.6244629891099108, 0.6376257216882676, 0.6376257216882676, 0.6376257216882676,
0.6542991471721685, 0.6542991471721685, 0.6542991471721685, 0.7033573442096076, 0.7033573442096076, 0.7033573442096076, 0.703911126676141, 0.703911126676141,
0.703911126676141, 0.7587414061130653, 0.7587414061130653, 0.7587414061130653, 0.8740076538936232, 0.8740076538936232, 0.8740076538936232, 0.884150425181996,
0.884150425181996, 0.884150425181996, 0.9426520776310289, 0.9426520776310289, 0.9426520776310289, 1.1282294239906596, 1.1282294239906596, 1.1282294239906596,
1.2153840648878125, 1.2153840648878125, 1.2153840648878125, 1.2079720982674649, 1.2079720982674649, 1.2079720982674649, 1.4132332099959584, 1.4132332099959584,
1.4132332099959584, 1.6230888667079653, 1.6230888667079653, 1.6230888667079653, 1.6410133756480851, 1.6410133756480851, 1.6410133756480851, 1.5881958805960414,
1.5881958805960414, 1.5881958805960414, 1.804646175017287, 1.804646175017287, 1.804646175017287, 1.8663031895199826, 1.8663031895199826, 1.8663031895199826,
1.722164272055739, 1.722164272055739, 1.722164272055739, 1.647652996618907, 1.647652996618907, 1.647652996618907, 1.6469506154771867, 1.6469506154771867,
1.6469506154771867, 1.5591307817064606, 1.5591307817064606, 1.5591307817064606, 1.4719108420425855, 1.4719108420425855, 1.4719108420425855, 1.4297892857851822,
1.4297892857851822, 1.4297892857851822, 1.2920953050015427, 1.2920953050015427, 1.2920953050015427, 1.2205711342919268, 1.2205711342919268, 1.2205711342919268,
1.1685208246563323, 1.1685208246563323, 1.1685208246563323, 1.049551130346437, 1.049551130346437, 1.049551130346437, 0.9610278990446985, 0.9610278990446985,
0.9610278990446985, 0.8977333939331735, 0.8977333939331735, 0.8977333939331735, 0.8334190606426094, 0.8334190606426094, 0.8334190606426094, 0.7648045083513764,
0.7648045083513764, 0.7648045083513764, 0.6988298101538835, 0.6988298101538835, 0.6988298101538835, 0.6585666878492391, 0.6585666878492391, 0.6585666878492391,
0.6111684996680828, 0.6111684996680828, 0.6111684996680828, 0.5665580244134178, 0.5665580244134178, 0.5665580244134178, 0.5148781211828849, 0.5148781211828849,
0.5148781211828849, 0.48618640994323403, 0.48618640994323403, 0.48618640994323403, 0.46033587532991155, 0.46033587532991155, 0.46033587532991155,
0.42457852975307236, 0.42457852975307236, 0.42457852975307236, 0.3910311549657173, 0.3910311549657173, 0.3910311549657173, 0.36425156818383675,
0.36425156818383675, 0.36425156818383675, 0.3470777453124518, 0.3470777453124518, 0.3470777453124518, 0.3297880637926003, 0.3297880637926003, 0.3297880637926003,
0.3107121988327585, 0.3107121988327585, 0.3107121988327585, 0.29652484531889745, 0.29652484531889745, 0.29652484531889745, 0.2811141469156768, 0.2811141469156768,
0.2811141469156768, 0.2667474285969589, 0.2667474285969589, 0.2667474285969589, 0.2617349980954065, 0.2617349980954065, 0.2617349980954065, 0.24441903266922857,
0.24441903266922857, 0.24441903266922857, 0.23718219453784634, 0.23718219453784634, 0.23718219453784634, 0.23607152333768355, 0.23607152333768355,
0.23607152333768355, 0.23895766208345, 0.23895766208345, 0.23895766208345, 0.23069303775657976, 0.23069303775657976, 0.23069303775657976, 0.22778154338144438,
0.22778154338144438, 0.22778154338144438, 0.22227276488421002, 0.22227276488421002, 0.22227276488421002, 0.2259470817454212, 0.2259470817454212,
0.2259470817454212, 0.2286492302134698, 0.2286492302134698, 0.2286492302134698, 0.23503432221840026, 0.23503432221840026, 0.23503432221840026,
0.23077484051921943, 0.23077484051921943, 0.23077484051921943, 0.2422018108014714, 0.2422018108014714, 0.2422018108014714, 0.2406218002553601, 0.2406218002553601,
0.2406218002553601, 0.26980269067502727, 0.26980269067502727, 0.26980269067502727, 0.2723817890491493, 0.2723817890491493, 0.2723817890491493,
0.29331623708078347, 0.29331623708078347, 0.29331623708078347, 0.33219796463591716, 0.33219796463591716, 0.33219796463591716, 0.3292209097084398,
0.3292209097084398, 0.3292209097084398, 0.39068358636774314, 0.39068358636774314, 0.39068358636774314, 0.45534117696074344, 0.45534117696074344,
0.45534117696074344, 0.49527403960628974, 0.49527403960628974, 0.49527403960628974, 0.5293983772269767, 0.5293983772269767, 0.5293983772269767,
0.5805258692936526, 0.5805258692936526, 0.5805258692936526, 0.5972556785462638, 0.5972556785462638, 0.5972556785462638, 0.6297278740840514, 0.6297278740840514,
0.6297278740840514, 0.6637066874967081, 0.6637066874967081, 0.6637066874967081, 0.7017438932271006, 0.7017438932271006, 0.7017438932271006, 0.6973996643075412,
0.6973996643075412, 0.6973996643075412, 0.6460060403539388, 0.6460060403539388, 0.6460060403539388, 0.6474303229677442, 0.6474303229677442, 0.6474303229677442,
0.6356648095891243, 0.6356648095891243, 0.6356648095891243, 0.5957781895580423, 0.5957781895580423, 0.5957781895580423, 0.5625754979314425, 0.5625754979314425,
0.5625754979314425, 0.5474479316706278, 0.5474479316706278, 0.5474479316706278, 0.5020326892714471, 0.5020326892714471, 0.5020326892714471, 0.4812812131075173,
0.4812812131075173, 0.4812812131075173, 0.4625050816019519, 0.4625050816019519, 0.4625050816019519, 0.4551534498168077, 0.4551534498168077, 0.4551534498168077,
0.41882891956888196, 0.41882891956888196, 0.41882891956888196, 0.40052291725759387, 0.40052291725759387, 0.40052291725759387, 0.3989206002929431,
0.3989206002929431, 0.3989206002929431, 0.3844624865835286, 0.3844624865835286, 0.3844624865835286, 0.3379402117997727, 0.3379402117997727, 0.3379402117997727])
```

```
        fetal_fractions_df = total_fetal_fraction * normalized_fetal_fractions_dist
        return(fetal_fractions_df)

def generate_length_distributions_plot(shared_lengths, fetal_lengths, fetal_sample):
        '''
        save the length distributions plot
        '''
        fetal_lengths_500 = fetal_lengths[fetal_lengths.index < 501]
        shared_lengths_500 = shared_lengths[shared_lengths.index < 501]
        shared_density = shared_lengths_500 / shared_lengths_500.sum()
        fetal_density = fetal_lengths_500 / fetal_lengths_500.sum()
        length_distributions_df = pd.concat([fetal_density.iloc[1:500], shared_density.iloc[1:500]], axis = 1)
        length_distributions_df.columns = ['fetal fragments', 'shared fragments']
        length_distributions_df.plot()
        plt.savefig(fetal_sample + '.length_distributions.png')

def calculate_total_fetal_fraction(shared_lengths, fetal_lengths):

        n_shared = int(shared_lengths.sum())
        n_fetal = int(fetal_lengths.sum())
        total_fetal_fraction = (2 * n_fetal) / (n_shared + n_fetal)

        return total_fetal_fraction

def create_fetal_fraction_per_length_df(shared_lengths, # pandas dataframe of shared fragments length distribution
                                      fetal_lengths, # pandas dataframe of fetal fragments length distribution
                                      total_fetal_fraction, # the total calculated percent of fetal DNA within the cfDNA
                                      err_rate, # TODO: will be later added to the model
                                      window = False, # for the length distributions. for example: 10 will give
                                      max_len = 500):
        '''
        output:
        make a dictionary that shows the fetal fraction at each fragment length
        input:
        shared_lengths - pandas dataframe of shared fragments length distribution
        fetal_lengths - pandas dataframe of fetal fragments length distribution
        total_fetal_fraction - the total calculated percent of fetal DNA within the cfDNA
        err_rate - TODO: will be later added to the model
        window - for the length distributions. for example: window=10 will give the same fetal fraction for fragments
        with length 0-10, 11-20, ...
        max_len - maximum fragment length used. default: 500.

        '''
        # TODO: rewrite a simpler version of this function


        # define bins (aka categories)
        bins = range(0, max_len, window)

        # make lists from two length distribution dataframes
        shared_lengths_list = []
        for i,c in shared_lengths.iterrows():
                for j in range(int(c)):
                        shared_lengths_list.append(int(i))

        fetal_lengths_list = []
        for i,c in fetal_lengths.iterrows():
                for j in range(int(c)):
                        fetal_lengths_list.append(int(i))

        # order the list using the bins - from a continuous variable to a categorical variable
        shared_pd_cut = pd.cut(shared_lengths_list, bins, include_lowest = True)
        fetal_pd_cut = pd.cut(fetal_lengths_list, bins, include_lowest = True)
        printverbose('shared_pd_cut')
        printverbose(shared_pd_cut)
        printverbose('fetal_pd_cut')
```

```
	printverbose(fetal_pd_cut)

	fetal_binned = pd.value_counts(fetal_pd_cut, sort = False).to_frame().values.tolist()
	shared_binned = pd.value_counts(shared_pd_cut, sort = False).to_frame().values.tolist()
	printverbose('fetal_binned')
	printverbose(fetal_binned)
	printverbose('shared_binned')
	printverbose(shared_binned)

	# for each bin, calculate its fetal fraction
	# if the fetal fraction is above 1 use (1-err);
	# if there is not enough fragments (under 5) use the result of the former window
	fetal_fraction_per_length_df = pd.Series(index = range(0, bins[-1] + 1, 1))

	binned_list_indices = [0] + list(nprepeat(range(len(fetal_binned)), window))

	for i in range(len(fetal_fraction_per_length_df)):
		idx_in_binned = binned_list_indices[i]
		fetal = fetal_binned[idx_in_binned][0]
		shared = shared_binned[idx_in_binned][0]
		if fetal > 5 or shared > 5: # TODO: or? and?
			ff = (2 * fetal) / (shared + fetal)
			if ff > 1:
				fetal_fraction_per_length_df[i] = 1 - err_rate
				# if i > 0:
				#	fetal_fraction_per_length_df[i] = fetal_fraction_per_length_df[i-1]
				# else:
				#	fetal_fraction_per_length_df[i] = fetal_fraction
			else:
				fetal_fraction_per_length_df[i] = ff
		else:
			if i > 0:
				fetal_fraction_per_length_df[i] = fetal_fraction_per_length_df[i-1]
			else:
				fetal_fraction_per_length_df[i] = total_fetal_fraction



	printverbose(fetal_fraction_per_length_df)

	return fetal_fraction_per_length_df

def calculate_err_rate():
	err = 0.003
	return err

def run_full_preprocessing(	db_path,
				fetal_sample,
				total_fetal_fraction,
				calculate_empirical_ff_dist,
				cores,
				window = False,
				max_len = 500,
				plot = False,
				qnames = False,
				region = False):

	printerr('pre-processing', 'calculating error rate (a place holder is temporarily set to 0.003)')
	err_rate = calculate_err_rate()

	calculate_fetal_fraction = not total_fetal_fraction

	if calculate_empirical_ff_dist or calculate_fetal_fraction:
		printerr('pre-processing', 'creating length distributions')
		shared_lengths, fetal_lengths = create_length_distributions(db_path, cores, qnames = qnames, region = region)
		if plot:
```

```
                printerr('pre-processing', 'saving length distributions plot as', fetal_sample + '.length_distributions.png')
                generate_length_distributions_plot(shared_lengths, fetal_lengths, fetal_sample)
        if calculate_fetal_fraction:
                printerr('pre-processing', 'calculating total fetal fraction')
                total_fetal_fraction = calculate_total_fetal_fraction(shared_lengths, fetal_lengths)

        printerr('total fetal fraction:', total_fetal_fraction)

        if calculate_empirical_ff_dist:
                printerr('pre-processing', 'calculating an empirical distribution of fetal fractions per fragment length')
                fetal_fractions_df = create_fetal_fraction_per_length_df(shared_lengths,
                                                                         fetal_lengths,
                                                                         total_fetal_fraction,
                                                                         err_rate,
                                                                         window,
                                                                         max_len = max_len)
        else:
                printerr('calculating an estimated distribution of fetal fractions per fragment length based on prior knowledge')
                fetal_fractions_df = estimate_length_distribution(total_fetal_fraction)



        return (err_rate, total_fetal_fraction, fetal_fractions_df)
```

stderr.py

```
from sys import stderr
from arguments import args as main_args

def printerr(output_to_print, *args, **kargs):
        print(output_to_print, file = stderr, sep = '\t', *args, **kargs)

def printverbose(output_to_print, *args, **kargs):
        if main_args.verbosity:
                print(output_to_print, file = stderr, *args, **kargs)
```

vcf_out.py

```
from collections import OrderedDict
import vcf
import parse_gt
import sys
from time import strftime
from stderr import *
import gzip

reserved_formats = ('GT', 'DP', 'AD', 'RO', 'QR', 'AO', 'QA', 'GL', 'PG', 'PP')

def print_info_or_format_row(info_or_format, field_id, number, field_type, description, source=False, output_path = False):
        line_list = []
        line_list.append('##' + info_or_format +'=<ID=' + field_id)
        line_list.append('Number=' + str(number)) # int, A, R, G, '.'
        line_list.append('Type=' + field_type)
        line_list.append('Description="' + description + '">')
        if source:
                line_list.append('Source="' + source + '"')

        printvcf(','.join(line_list), out_path = output_path)

def printvcf(x, *args, out_path = False, **kargs):
        if out_path:
                with open(out_path, 'a') as f:
                        print(x, file = f, *args, **kargs)
        else:
                print(x, *args, **kargs)

def make_header(cfdna_vcf_reader,
```

```
            parents_vcf_reader,
            input_command,
            total_fetal_fraction,
            fetal_sample_name,
            reserved_formats,
            output_path = False):

    if cfdna_vcf_reader.metadata['reference'] != parents_vcf_reader.metadata['reference']:
            printerr('Warning! are the vcf files based on the same reference genome?')
    if cfdna_vcf_reader.contigs != parents_vcf_reader.contigs:
            printerr('Warning! cfdna and parental vcf files have different contigs')

    # print unique header fields
    printvcf(       '##fileformat=' + cfdna_vcf_reader.metadata['fileformat'],
                    '##fileDate=' + strftime('%Y%m%d'),
                    '##source=hoobari',
                    '##phasing=none', # phasing is not yet supported
                    '##reference=' + cfdna_vcf_reader.metadata['reference'],
                    '##commandline="' + input_command + '"',
                    '##fetalfraction=' + str(total_fetal_fraction),
                    sep = '\n',
                    out_path = output_path)

    # print contigs header fields
    cfdna_contigs_output = []
    for c in cfdna_vcf_reader.contigs.values():
            cfdna_contigs_output.append('##contig=<ID=' + str(c.id) + ',length=' + str(c.length) + '>')
    if len(cfdna_contigs_output) > 0:
            printvcf('\n'.join(cfdna_contigs_output), out_path = output_path)

    # TODO: print filter header fields from parents?

    # print format and info header fields
    printvcf(
    '##FORMAT=<ID=GT,Number=1,Type=String,Description="Genotype",Source="hoobari">',
    '##FORMAT=<ID=DP,Number=1,Type=String,Description="Read Depth">',
    '##FORMAT=<ID=AD,Number=R,Type=String,Description="Number of observation for each allele">',
    '##FORMAT=<ID=RO,Number=1,Type=String,Description="Reference allele observation count">',
    '##FORMAT=<ID=QR,Number=1,Type=String,Description="Sum of quality of the reference observations">',
    '##FORMAT=<ID=AO,Number=A,Type=String,Description="Alternate allele observation count">',
    '##FORMAT=<ID=QA,Number=A,Type=String,Description="Sum of quality of the alternate observations">',
    '##FORMAT=<ID=GL,Number=G,Type=Float,Description="Genotype Likelihood, log10-scaled likelihoods of the data given the called genotype for each possible
genotype generated from the reference and alternate alleles given the sample ploidy",Source="hoobari">',
    '##FORMAT=<ID=PG,Number=G,Type=Float,Description="P(Genotype), Per-site genotype prior probabilities",Source="hoobari">',
    '##FORMAT=<ID=PP,Number=G,Type=Float,Description="P(Posterior), Per-site genotype posterior probabilities",Source="hoobari">',
    '##INFO=<ID=MGT,Number=1,Type=String,Description="Mother\' Genotype">',
    '##INFO=<ID=MGQ,Number=1,Type=Float,Description="Mother\'s Genotype Quality, the Phred-scaled marginal (or unconditional) probability of the called
genotype">',
    '##INFO=<ID=MGL,Number=G,Type=Float,Description="Mother\'s Genotype Likelihood, log10-scaled likelihoods of the data given the called genotype for each
possible genotype generated from the reference and alternate alleles given the sample ploidy">',
    '##INFO=<ID=MAD,Number=R,Type=Integer,Description="Mother\'s Number of observation for each allele">',
    '##INFO=<ID=MDP,Number=1,Type=Integer,Description="Mother\'s Read Depth">',
    '##INFO=<ID=MRO,Number=1,Type=Integer,Description="Mother\'s Reference allele observation count">',
    '##INFO=<ID=MQR,Number=1,Type=Integer,Description="Mother\'s Sum of quality of the reference observations">',
    '##INFO=<ID=MAO,Number=A,Type=Integer,Description="Mother\'s Alternate allele observation count">',
    '##INFO=<ID=MQA,Number=A,Type=Integer,Description="Mother\'s Sum of quality of the alternate observations">',
    '##INFO=<ID=FGT,Number=1,Type=String,Description="Father\'s Genotype">',
    '##INFO=<ID=FGQ,Number=1,Type=Float,Description="Father\'s Genotype Quality, the Phred-scaled marginal (or unconditional) probability of the called
genotype">',
    '##INFO=<ID=FGL,Number=G,Type=Float,Description="Father\'s Genotype Likelihood, log10-scaled likelihoods of the data given the called genotype for each
possible genotype generated from the reference and alternate alleles given the sample ploidy">',
    '##INFO=<ID=FAD,Number=R,Type=Integer,Description="Father\'s Number of observation for each allele">',
    '##INFO=<ID=FDP,Number=1,Type=Integer,Description="Father\'s Read Depth">',
    '##INFO=<ID=FRO,Number=1,Type=Integer,Description="Father\'s Reference allele observation count">',
    '##INFO=<ID=FQR,Number=1,Type=Integer,Description="Father\'s Sum of quality of the reference observations">',
    '##INFO=<ID=FAO,Number=A,Type=Integer,Description="Father\'s Alternate allele observation count">',
```

```
        '##INFO=<ID=FQA,Number=A,Type=Integer,Description="Father\'s Sum of quality of the alternate observations">',
        '##INFO=<ID=MFQ,Number=1,Type=Float,Description="Mother\'s and Father\'s QUAL score from the parental vcf">',
        sep = '\n',
        out_path = output_path)

        # get cfdna vcf infos header
        cfdna_vcf_compressed = cfdna_vcf_reader.filename.endswith('.gz')
        if cfdna_vcf_compressed:
                f = gzip.open(cfdna_vcf_reader.filename, 'rb')
                cfdna_vcf_infos_list = [line.decode().strip() for line in f if line.decode().startswith('##INFO')]
        else:
                f = open(cfdna_vcf_reader.filename, 'r')
                cfdna_vcf_infos_list = [line.strip() for line in f if line.startswith('##INFO')]
        f.close()
        # get parents vcf infos header
        parents_vcf_compressed = parents_vcf_reader.filename.endswith('.gz')
        if parents_vcf_compressed:
                f = gzip.open(parents_vcf_reader.filename, 'rb')
                parents_vcf_infos_list = [line.decode().strip() for line in f if line.decode().startswith('##INFO')]
        else:
                f = open(parents_vcf_reader.filename, 'r')
                parents_vcf_infos_list = [line.strip() for line in f if line.startswith(b'##INFO')]
        f.close()
        parents_vcf_infos_list = [l.replace('ID=','ID=P') for l in parents_vcf_infos_list]
        parents_vcf_infos_list = [l.replace('Description="','Description="Parents ') for l in parents_vcf_infos_list]

        printvcf('\n'.join(cfdna_vcf_infos_list), sep = '\n', out_path = output_path)
        printvcf('\n'.join(parents_vcf_infos_list), sep = '\n', out_path = output_path)

        # print column names
        vcf_columns = ['#CHROM','POS','ID','REF','ALT','QUAL','FILTER','INFO','FORMAT'] + [fetal_sample_name]
        printvcf('\t'.join(vcf_columns), out_path = output_path)

def rec_sample_to_string(rec, sample):
        data = rec.genotype(sample).data
        #print(data)

        if data and data.GT != '.':
                format_and_gt_dic = OrderedDict([])
                format_list = rec.FORMAT.split(':')
                for f in format_list:
                        idx = format_list.index(f)
                        if f in ('AD', 'GL'):
                                value = ','.join(str(i) for i in data[idx])
                        elif type(data[idx]) is list:
                                value = ','.join([str(i) for i in data[idx]])
                        else:
                                value = str(data[idx])

                        format_and_gt_dic[f] = value
        else:
                format_and_gt_dic = '.'

        return format_and_gt_dic

def parents_gt_to_info(mother_id, father_id, parents_rec):
        rec_info_list = []
        for parent_sample in (mother_id, father_id):
                for field in parents_rec.FORMAT.split(':'):
                        if parent_sample == mother_id:
                                prefix = 'M'
                        elif parent_sample == father_id:
                                prefix = 'F'
                        parents_sample_field_data = parents_rec.genotype(parent_sample)[field]
                        if type(parents_sample_field_data) is list: # some fields contain a few values
                                parents_sample_field_data = ','.join([str(i) for i in parents_sample_field_data])
```

```
                    rec_info_list.append(prefix + field + '=' + str(parents_sample_field_data))
        rec_info_list.append('MFQ=' + str(parents_rec.QUAL))
        return ';'.join(rec_info_list)

def info_to_string(info_dic):
        rec_info_list = []
        for field in info_dic:
                field_data = info_dic[field]
                if type(field_data) is list:
                        field_data = ','.join([str(i) for i in field_data])
                rec_info_list.append(field + '=' + str(field_data))
        return ';'.join(rec_info_list)

def print_var(rec, phred, pos_info, format_and_gt_dic, out_path = False):

        row_list = []

        # columns 1 - 5
        row_list += [rec.CHROM, str(rec.POS), '.', rec.REF, str(rec.ALT[0])]

        # column 6-7
        row_list += [str(phred), '.']

        # column 8
        # info_list = [str(k) + '=' + str(pos_info_dic[k]) for k in pos_info_dic]
        # row_list += [';'.join(info_list)]
        row_list += [pos_info]

        # columns 9-10
        if format_and_gt_dic == '.':
                row_list += [':'.join(reserved_formats), '.']
        else:
                format_list = list(format_and_gt_dic.keys())
                fetal_gt_list = list(format_and_gt_dic.values())
                row_list += [':'.join(format_list)] + [':'.join(fetal_gt_list)]

        # merge all to one row string
        variant_row = '\t'.join(row_list)

        printvcf(variant_row, out_path = out_path)

def unsupported_position(rec, out_path = False):

                alt = ','.join([str(i) for i in rec.ALT])

                variant_row = [        rec.CHROM,
                                str(rec.POS),
                                '.',
                                rec.REF,
                                alt,
                                '0',
                                '.',
                                '.',
                                ':'.join(reserved_formats),
                                '.']

                printvcf('\t'.join(variant_row), out_path = out_path)
```

vcfuid.py

```
from re import split as regsplit

def rec_to_uid(rec):
        uid = rec.CHROM + ':' + str(rec.POS) + '_' + rec.REF + '/' + str(rec.ALT[0])
        return uid
```

```
def uid_to_rec(uid):
        rec = regsplit(r':|_|/', uid)
        return rec
```

accuracy_evaluation_indels_trained_on_snpindels.py


```
# BSD 3-Clause License
# Copyright (c) 2019 Noam Shomron, Tom Rabinowitz
# All rights reserved.
# Redistribution and use in source and binary forms, with or without modification, are permitted provided that the following conditions are met:
# * Redistributions of source code must retain the above copyright notice, this list of conditions and the following disclaimer.
# * Redistributions in binary form must reproduce the above copyright notice, this list of conditions and the following disclaimer in the documentation and/or
#   other materials provided with the distribution.
# * Neither the name of the copyright holder nor the names of its contributors may be used to endorse or promote products derived from this software without
#   specific prior written permission.
# THIS SOFTWARE IS PROVIDED BY THE COPYRIGHT HOLDERS AND CONTRIBUTORS "AS IS" AND ANY EXPRESS OR IMPLIED WARRANTIES, INCLUDING, BUT NOT LIMITED TO, THE IMPLIED
# WARRANTIES OF MERCHANTABILITY AND FITNESS FOR A PARTICULAR PURPOSE ARE DISCLAIMED. IN NO EVENT SHALL THE COPYRIGHT HOLDER OR CONTRIBUTORS BE LIABLE FOR ANY
# DIRECT, INDIRECT, INCIDENTAL, SPECIAL, EXEMPLARY, OR CONSEQUENTIAL DAMAGES (INCLUDING, BUT NOT LIMITED TO, PROCUREMENT OF SUBSTITUTE GOODS OR SERVICES; LOSS OF
# USE, DATA, OR PROFITS; OR BUSINESS INTERRUPTION) HOWEVER CAUSED AND ON ANY THEORY OF LIABILITY, WHETHER IN CONTRACT, STRICT LIABILITY, OR TORT (INCLUDING
# NEGLIGENCE OR OTHERWISE) ARISING IN ANY WAY OUT OF THE USE OF THIS SOFTWARE, EVEN IF ADVISED OF THE POSSIBILITY OF SUCH DAMAGE.
```


```
import sys
import warnings
warnings.filterwarnings('ignore')
from sklearn.metrics import roc_curve, auc, accuracy_score, precision_recall_fscore_support

import xgboost as xgb
from sklearn.utils import class_weight
from sklearn.cross_validation import KFold
from sklearn.multiclass import OneVsRestClassifier
from sklearn.preprocessing import label_binarize, scale
from sklearn.ensemble import VotingClassifier, BaggingClassifier


# FEATURES
FEATURES_BASIC = ['CHROM', 'POS', 'REF', 'ALT']
FEATURES_HOOBARI = ['QUAL', 'GT', 'PP_homref', 'PP_het', 'PP_homalt', 'PG_homref', 'PG_het', 'PG_homalt',
                                    'GL_homref', 'GL_het', 'GL_homalt']
FEATURES_CFDNA_FREEBAYES = ['AB', 'DP', 'GTI', 'RPL', 'AF', 'SAP',  'EPP', 'QR', 'MQMR', 'SRR', 'PQR', 'PQA', 'PAO', 'PRO', 'TYPE',
                                                    'AO', 'SAR', 'EPPR', 'SRP', 'MEANALT', 'PAIREDR', 'technology_ILLUMINA', 'RPR', 'MQM', 'RPPR', 'RPP',
                                                    'AC', 'NS', 'PAIRED', 'SRF', 'SAF', 'DPB', 'NUMALT', 'RO', 'ABP', 'ODDS', 'QA', 'LEN','CIGAR', 'RUN']
FEATURES_MATERNAL = ['MQA', 'MRO', 'MGT', 'MDP', 'MGL_homref', 'MGL_het', 'MGL_homalt', 'MAO', 'MQR']
FEATURES_PATERNAL = ['FQA', 'FRO', 'FGT', 'FDP', 'FGL_homref', 'FGL_het', 'FGL_homalt', 'FAO', 'FQR']
FEATURES_PARENTS = ['PAN', 'PODDS', 'PEPPR', 'PLEN', 'PTYPE', 'PAC', 'PRPPR', 'PPAO', 'PRPP', 'PAF',
                                     'PSRR', 'PRPR', 'PABP', 'PSAR', 'PNUMALT', 'PMEANALT', 'PGTI', 'PPQA', 'PMQM',
                                     'PPRO', 'PSRP', 'PDP', 'PAB', 'PPAIREDR', 'PPQR', 'PDPB', 'PSRF', 'PCIGAR', 'PPAIRED',
                                     'PDPRA', 'PSAP', 'PMQMR', 'Ptechnology_ILLUMINA', 'MFQ', 'PRPL', 'PSAF', 'PEPP', 'PNS', 'PRUN']
ALL_FEATURES = list(set(FEATURES_BASIC + FEATURES_HOOBARI + FEATURES_CFDNA_FREEBAYES + FEATURES_MATERNAL +
                                                    FEATURES_PATERNAL + FEATURES_PARENTS))

# QUERIES
FAMILY_DATA_QUERY = """
SELECT {}
  FROM merged
 WHERE CHROM not in ("chrX","chrY")
   AND not (PG_homref=0.33333 and PG_het=0.33333 and PG_homalt=0.33333)
   AND GT in ("0/0","0/1","1/1")
   AND t_GT in ("0/0","0/1","1/1")
   AND TYPE {}
   AND {}
"""
VAR_TYPES = {'indel': ' in ("ins","del")'}
TRAIN_OPTIONS = {'MhetFhet positions': 'MGT="0/1" and FGT="0/1"',\
                              'MhetFhom positions': 'MGT="0/1" and FGT in ("0/0","1/1")',\
                              'FhetMhom positions': 'FGT="0/1" and MGT in ("0/0","1/1")'}\
```

```
TEST_OPTIONS = {'MhetFhet positions': 'MGT="0/1" and FGT="0/1"',\
                              'MhetFhom positions': 'MGT="0/1" and FGT in ("0/0","1/1")',\
                              'FhetMhom positions': 'FGT="0/1" and MGT in ("0/0","1/1")'}

# TRAIN_TEST
TEST_FAMILY = "G2"
CLASS_COLUMN = "t_GT"
FAMILIES = ["G1", "G2"]
DBS_DIRECTORY = "/path/to/directory/"
RESULTS_PATH = "/path/to/directory/acc_evaluation_g2_wgs_only_wes_last.xlsx"

# ML MODELS
#CLASSIFIERS = [ExtraTreesClassifier(random_state=42, n_estimators=100)]

CLASSIFIERS = [RandomForestClassifier(random_state=42, n_estimators=100, n_jobs=-1),
                         AdaBoostClassifier(random_state=42, n_estimators=100),
                         xgb.sklearn.XGBClassifier(n_estimators=100, random_state=42, n_jobs=-1),
                         KNeighborsClassifier(),
                         LogisticRegression(random_state=42),
                         VotingClassifier(estimators=[("rf", RandomForestClassifier(random_state=42, n_estimators=100, n_jobs=-1)),
                                                                    ('ada',AdaBoostClassifier(random_state=42, n_estimators=100)),
                                                                    ('xgb', xgb.sklearn.XGBClassifier(random_state=42, n_estimators=100))], voting='soft')]


#TRAIN AND TEST DATA CREATION FUNCTIONS
def sqllite_shlif(family, query):
       """
       gets family id and returns pd of family data
       """
       if family == 'G2':
              con = sqlite3.connect(DBS_DIRECTORY + family + "_wgs_TRAIN_wes.db")
       elif family == "G1":
              con = sqlite3.connect(DBS_DIRECTORY + family + "_wes.db")
       df = pd.read_sql_query(query, con)
       return df

def train_test_data_creator(families_names, columns, variant_type, parents_settings):
       """
       """
       families_dfs = []
       for family in families_names:
              f_df = sqllite_shlif(family, FAMILY_DATA_QUERY.format(",".join(columns),\
                                                                                      variant_type,\
                                                                                      parents_settings))
              families_dfs.append(f_df)
       return pd.concat(families_dfs).drop_duplicates()

#PREPROCESSING FUNCTIONS
def cigar_len(cigar):
       return sum([int(s) for s in re.findall('\d+', cigar)])

def indel_len(row):
       return abs(len(row.REF) - len(row.ALT))

def indel_base_count(row, base):
       if row.TYPE in ('del', 'ins'):
              if len(row.REF)> len(row.ALT):
                     bases = list(row.REF)
                     for b in row.ALT:
                            bases.remove(b)
                     return bases.count(base)
              else:
                     bases = list(row.ALT)
                     for b in row.REF:
                            bases.remove(b)
                     return bases.count(base)
```

```
        else:
                return 0

def len_alphabetic_category_order(column_data):
        """
        """
        order = list(set(column_data))
        order.sort()
        order.sort(key=len)
        return order

def preprocess_data(x, remove_bad_values=False):
        """
        """
        fetures_to_preprocess = x.select_dtypes(['object']).columns
        if 'CIGAR_LEN' in fetures_to_preprocess:
                x['CIGAR_LEN'] = x.apply(lambda x: cigar_len(x.CIGAR), axis=1)
        if 'PCIGAR_LEN' in fetures_to_preprocess:
                x['PCIGAR_LEN'] = x.apply(lambda x: cigar_len(x.PCIGAR), axis=1)
        if 'TYPE' in fetures_to_preprocess and ('ins' in set(x['TYPE']) or 'del' in set(x['TYPE'])) \
                and 'ALT' in fetures_to_preprocess and 'REF' in fetures_to_preprocess:
                        x['INDEL_LEN'] = x.apply(indel_len, axis=1)
                        x['INDEL_A_COUNTS'] = x.apply(lambda x: indel_base_count(x, 'A'), axis=1)
                        x['INDEL_T_COUNTS'] = x.apply(lambda x: indel_base_count(x, 'T'), axis=1)
                        x['INDEL_G_COUNTS'] = x.apply(lambda x: indel_base_count(x, 'G'), axis=1)
                        x['INDEL_C_COUNTS'] = x.apply(lambda x: indel_base_count(x, 'C'), axis=1)
        if 'GT' in fetures_to_preprocess:
                x['GT'] = x['GT'].map({'0/0': 0, '0/1': 1, '1/1': 2}).astype(float)
        if 'FGT' in fetures_to_preprocess:
                x['FGT'] = x['FGT'].map({'0/0': 0, '0/1': 1, '1/1': 2}).astype(float)
        if 'MGT' in fetures_to_preprocess:
                x['MGT'] =  x['MGT'].map({'0/0': 0, '0/1': 1, '1/1': 2}).astype(float)
        if 't_GT' in fetures_to_preprocess:
                x['t_GT'] =  x['t_GT'].map({'0/0': 0, '0/1': 1, '1/1': 2}).astype(float)
        if 'TYPE' in fetures_to_preprocess:
                x['TYPE'] = x['TYPE'].map({'del': 0, 'ins' : 1, 'snp' : 2})
        if 'PTYPE' in fetures_to_preprocess:
                x['PTYPE'] = x['PTYPE'].map({'del': 0, 'ins' : 1, 'snp' : 2,\
                                                                     'complex': 3, 'mnp': 4})
        if 'CIGAR' in fetures_to_preprocess:
                x['CIGAR'] = x['CIGAR'].astype("category",\
                                                                        categories=len_alphabetic_category_order(x['CIGAR'])).cat.codes
        if 'PCIGAR' in fetures_to_preprocess:
                x['PCIGAR'] = x['PCIGAR'].astype("category",\
                                                                        categories=len_alphabetic_category_order(x['PCIGAR'])).cat.codes
        if 'ALT' in fetures_to_preprocess:
                x['ALT'] = x['ALT'].astype("category",\
                                                                        categories=len_alphabetic_category_order(x['ALT'])).cat.codes
        if 'REF' in fetures_to_preprocess:
                x['REF'] = x['REF'].astype("category",\
                                                                categories=len_alphabetic_category_order(x['REF'])).cat.codes
        if 'CHROM' in fetures_to_preprocess:
                x['CHROM'] = x['CHROM'].map({'chr1': 0, 'chr2': 1, 'chr3': 2, 'chr4': 3, 'chr5': 4,\
                                                                      'chr6': 5, 'chr7': 6, 'chr8': 7, 'chr9': 8, 'chr10': 9,\
                                                                      'chr11': 10, 'chr12': 11, 'chr13': 12, 'chr14': 13, 'chr15': 14,\
                                                                      'chr16': 15, 'chr17': 16, 'chr18': 17, 'chr19': 18, 'chr20': 19,\
                                                                      'chr21': 20, 'chr22': 21}).astype(float)

        if remove_bad_values:
                bad_values_indexes = x[((x.MGT == 1.0) & (x.FGT == 2.0) & (x.t_GT == 0.0)) |\
                                                      ((x.MGT == 2.0) & (x.FGT == 1.0) & (x.t_GT == 0.0)) |\
                                                      ((x.MGT == 2.0) & (x.FGT == 2.0) & (x.t_GT != 2.0)) |\
                                                      ((x.MGT == 0.0) & (x.FGT == 0.0) & (x.t_GT != 0.0))].index
                x.drop(bad_values_indexes)
        return x
```

```
#MODEL EVALUATION FUNCTIONS
def average_accuracy_score(y_test, acc_prediction, classes):
        all_acc = []
        r = ""
        for c in classes:
                c_acc= accuracy_score(y_test[y_test==c], \
                                                        acc_prediction[pd.Series(y_test)[y_test==c].index])
                r += "{} accuracy-{}\n".format(c, round(c_acc, 2))
                all_acc.append(c_acc)
        r += "averaged accuray-{}".format(round(sum(all_acc)/len(all_acc),2))
        return r

def hoobari_model_scores(train_data, test_data, clf):
        """
        """
        p_train_data = preprocess_data(train_data)
        p_test_data = preprocess_data(test_data)
        X_test = p_test_data[ALL_FEATURES].values
        y_test = p_test_data[CLASS_COLUMN].values
        X_train = p_train_data[ALL_FEATURES].values
        y_train = p_train_data[CLASS_COLUMN].values
        classes = sorted(set(y_train))

        clf.fit(X_train, y_train)
        acc_prediction = clf.predict(X_test)

        accuraccy = accuracy_score(y_test, acc_prediction)
        average_accuracy = average_accuracy_score(y_test, acc_prediction, classes)

        return accuraccy, average_accuracy

def auc_score(clf, train_data, test_data):
        p_train_data = preprocess_data(train_data)
        p_test_data = preprocess_data(test_data)
        x_train = p_train_data[ALL_FEATURES].values
        y_train = p_train_data[CLASS_COLUMN].values
        x_test = p_test_data[ALL_FEATURES].values
        y_test = p_test_data[CLASS_COLUMN].values
        if clf.__class__.__name__ != 'XGBClassifier':
                classifier = OneVsRestClassifier(clf)
        else:
                classifier = OneVsRestClassifier(xgb.sklearn.XGBClassifier(random_state=42, n_jobs=-1))
        classifier.fit(x_train, y_train)
        b_y_test =  label_binarize(y_test, classes=[0, 1, 2])
        try:
                y_score = classifier.predict_proba(x_test)
        except:
                print(clf.__class__.__name__)
                print("desicion function")
                y_score = classifier.desicion_function(x_test)

        n_classes = b_y_test.shape[1]
        fpr = dict()
        tpr = dict()
        roc_auc = dict()
        for i in range(n_classes):
                fpr[i], tpr[i], _ = roc_curve(b_y_test[:, i], y_score[:, i])
                roc_auc[i] = auc(fpr[i], tpr[i])
        # Compute micro-average ROC curve and ROC area
        fpr["micro"], tpr["micro"], _ = roc_curve(b_y_test.ravel(), y_score.ravel())
        roc_auc["micro"] = auc(fpr["micro"], tpr["micro"])
        return roc_auc["micro"]

def hoob_auc_score(train_data, test_data):
        p_train_data = preprocess_data(train_data)
        p_test_data = preprocess_data(test_data)
```

```
        x_train = p_train_data[ALL_FEATURES].values
        y_train = p_train_data[CLASS_COLUMN].values
        x_test = p_test_data[ALL_FEATURES].values
        y_test = p_test_data[CLASS_COLUMN].values
        b_y_test =  label_binarize(y_test, classes=[0, 1, 2])
        n_classes = b_y_test.shape[1]
        y_score = np.array(test_data[['PP_homref', 'PP_het', 'PP_homalt']].reset_index(drop=True))
        fpr = dict()
        tpr = dict()
        roc_auc = dict()
        for i in range(n_classes):
                fpr[i], tpr[i], _ = roc_curve(b_y_test[:, i], y_score[:, i])
                roc_auc[i] = auc(fpr[i], tpr[i])

        # Compute micro-average ROC curve and ROC area
        fpr["micro"], tpr["micro"], _ = roc_curve(b_y_test.ravel(), y_score.ravel())
        roc_auc["micro"] = auc(fpr["micro"], tpr["micro"])
        return roc_auc["micro"]
def main():
        """
        [test_family, Type(indel/snp), model, features, parameters, trained_on,\
         parents_test_settings, ml_acc, ml_auc, hoobari_acc, hoobari_auc]
        """
        results = []
        #for test_family in FAMILIES:
        for var_type in VAR_TYPES:
                train_var_type_query = ' in ("ins", "del", "snp")'
                test_var_type_query = VAR_TYPES[var_type]
                for test_option in TEST_OPTIONS:
                        test_query = TEST_OPTIONS[test_option]
                        train_query = test_query
                        train_data = train_test_data_creator([f for f in FAMILIES if f != TEST_FAMILY],\
                                                                        ALL_FEATURES + [CLASS_COLUMN],\
                                                                        train_var_type_query, train_query)
                        parents_test_settings = test_option
                        test_data = train_test_data_creator([TEST_FAMILY], ALL_FEATURES + [CLASS_COLUMN],\
                                                                                    test_var_type_query, test_query)
                        trained_on = ",".join([f for f in FAMILIES if f != TEST_FAMILY]) + "\n" + train_query

                        for clf in CLASSIFIERS:
                                clf_name = name = clf.__class__.__name__
                                model_acc, model_avg_acc = hoobari_model_scores(train_data, test_data, clf)
                                hoob_acc = accuracy_score(test_data['t_GT'].values, test_data['GT'].values)
                                hoob_avg_acc = average_accuracy_score(test_data['t_GT'].values, test_data['GT'].values,\
                                 sorted(set(test_data['t_GT'].values)))
                                hoob_auc = hoob_auc_score(train_data, test_data)
                                test_class_balance = test_data['t_GT'].value_counts(normalize=True)
                                train_class_balance = train_data['t_GT'].value_counts(normalize=True)
                                micro_auc = auc_score(clf, train_data, test_data)
                                results.append([TEST_FAMILY, var_type, clf_name, parents_test_settings,\
                                                              hoob_acc, hoob_avg_acc, hoob_auc, model_acc, model_avg_acc, micro_auc,\
                                                              trained_on, test_class_balance, train_class_balance])
        results_df = pd.DataFrame(results, columns=['test_family', 'var_type', 'clf_name', 'parents_test_settings',\
                                                                                    'hoob_acc', 'hoob_avg_acc', 'hoob_auc', 'model_acc', 'model_avg_acc',
'model_micro_auc',\
                                                                                    'trained_on', 'test_class_balance', 'train_class_balance'])
        results_df.to_excel(RESULTS_PATH)


if __name__ == '__main__':
        main()
```

final_results_check_with_snpindel_train_on_test_settings.py

```
import sys
```

```
import warnings
warnings.filterwarnings('ignore')
from sklearn.metrics import roc_curve, auc, accuracy_score, precision_recall_fscore_support

import pickle
import xgboost as xgb
from sklearn.utils import class_weight
from sklearn.cross_validation import KFold
from sklearn.multiclass import OneVsRestClassifier
from sklearn.preprocessing import label_binarize, scale
from sklearn.ensemble import VotingClassifier, BaggingClassifier
from sklearn.calibration import calibration_curve, CalibratedClassifierCV
import matplotlib.pyplot as plt
plt.switch_backend('agg')


# FEATURES
FEATURES_BASIC = ['CHROM', 'POS', 'REF', 'ALT']
FEATURES_HOOBARI = ['QUAL', 'GT', 'PP_homref', 'PP_het', 'PP_homalt', 'PG_homref', 'PG_het', 'PG_homalt',
                                     'GL_homref', 'GL_het', 'GL_homalt']
FEATURES_CFDNA_FREEBAYES = ['AB', 'DP', 'GTI', 'RPL', 'AF', 'SAP',  'EPP', 'QR', 'MQMR', 'SRR', 'PQR', 'PQA', 'PAO', 'PRO', 'TYPE',
                                                    'AO', 'SAR', 'EPPR', 'SRP', 'MEANALT', 'PAIREDR', 'technology_ILLUMINA', 'RPR', 'MQM', 'RPPR', 'RPP',
                                                    'AC', 'NS', 'PAIRED', 'SRF', 'SAF', 'DPB', 'NUMALT', 'RO', 'ABP', 'ODDS', 'QA', 'LEN','CIGAR', 'RUN']
FEATURES_MATERNAL = ['MQA', 'MRO', 'MGT', 'MDP', 'MGL_homref', 'MGL_het', 'MGL_homalt', 'MAO', 'MQR']
FEATURES_PATERNAL = ['FQA', 'FRO', 'FGT', 'FDP', 'FGL_homref', 'FGL_het', 'FGL_homalt', 'FAO', 'FQR']
FEATURES_PARENTS = ['PAN', 'PODDS', 'PEPPR', 'PLEN', 'PTYPE', 'PAC', 'PRPPR', 'PPAO', 'PRPP', 'PAF',
                                     'PSRR', 'PRPR', 'PABP', 'PSAR', 'PNUMALT', 'PMEANALT', 'PGTI', 'PPQA', 'PMQM',
                                     'PPRO', 'PSRP', 'PDP', 'PAB', 'PPAIREDR', 'PPQR', 'PDPB', 'PSRF', 'PCIGAR', 'PPAIRED',
                                     'PDPRA', 'PSAP', 'PMQMR', 'Ptechnology_ILLUMINA', 'MFQ', 'PRPL', 'PSAF', 'PEPP', 'PNS', 'PRUN']
ALL_FEATURES = list(set(FEATURES_BASIC + FEATURES_HOOBARI + FEATURES_CFDNA_FREEBAYES + FEATURES_MATERNAL +
                                                    FEATURES_PATERNAL + FEATURES_PARENTS))

# QUERIES
FAMILY_DATA_QUERY = """
SELECT {}
  FROM merged
 WHERE CHROM not in ("chrX","chrY")
   AND not (PG_homref=0.33333 and PG_het=0.33333 and PG_homalt=0.33333)
   AND GT in ("0/0","0/1","1/1")
   AND t_GT in ("0/0","0/1","1/1")
   AND TYPE {}
   AND {}
"""
VAR_TYPES = {'snp': '="snp"',\
                        'indel': ' in ("ins","del")'}
TRAIN_OPTIONS = {'MhetFhet positions': 'MGT="0/1" and FGT="0/1"',\
                              'MhetFhom positions': 'MGT="0/1" and FGT in ("0/0","1/1")',\
                              'FhetMhom positions': 'FGT="0/1" and MGT in ("0/0","1/1")'}\


TEST_OPTIONS = {'MhetFhet positions': 'MGT="0/1" and FGT="0/1"',\
                              'MhetFhom positions': 'MGT="0/1" and FGT in ("0/0","1/1")',\
                              'FhetMhom positions': 'FGT="0/1" and MGT in ("0/0","1/1")'}

# TRAIN_TEST
TEST_TRAIN_FAMILIES = {"G2_wgs_TEST": ["G1_wgs"],
                                        "G5_wgs": ["G1_wgs", "G2_wgs"]}
CLASS_COLUMN = "t_GT"
FAMILIES = ["G1", "G2"]
DBS_DIRECTORY = "/path/to/dir/" #directory should contain sqlite3 versions of VCF files
RESULTS_PATH = "/path/to/dir/acc_evaluation_g2_final_wgs.xlsx"

CLASSIFIER = RandomForestClassifier(random_state=42, n_estimators=100, n_jobs=-1)

#TRAIN AND TEST DATA CREATION FUNCTIONS
def sqllite_shlif(family, query):
```

```
	"""
	gets family id and returns pd of family data
	"""
	con = sqlite3.connect(DBS_DIRECTORY + family + ".db")
	df = pd.read_sql_query(query, con)
	return df

def train_test_data_creator(families_names, columns, variant_type, parents_settings):
	"""
	"""
	families_dfs = []
	for family in families_names:
		f_df = sqllite_shlif(family, FAMILY_DATA_QUERY.format(",".join(columns),\
									variant_type,\
									parents_settings))
		families_dfs.append(f_df)
	return pd.concat(families_dfs).drop_duplicates()

#PREPROCESSING FUNCTIONS
def cigar_len(cigar):
	return sum([int(s) for s in re.findall('\d+', cigar)])

def indel_len(row):
	return abs(len(row.REF) - len(row.ALT))

def indel_base_count(row, base):
	if row.TYPE in ('del', 'ins'):
		if len(row.REF)> len(row.ALT):
			bases = list(row.REF)
			for b in row.ALT:
				bases.remove(b)
			return bases.count(base)
		else:
			bases = list(row.ALT)
			for b in row.REF:
				bases.remove(b)
			return bases.count(base)
	else:
		return 0

def len_alphabetic_category_order(column_data):
	"""
	"""
	order = list(set(column_data))
	order.sort()
	order.sort(key=len)
	return order

def preprocess_data(x, remove_bad_values=False):
	"""
	"""
	fetures_to_preprocess = x.select_dtypes(['object']).columns
	if 'CIGAR_LEN' in fetures_to_preprocess:
		x['CIGAR_LEN'] = x.apply(lambda x: cigar_len(x.CIGAR), axis=1)
	if 'PCIGAR_LEN' in fetures_to_preprocess:
		x['PCIGAR_LEN'] = x.apply(lambda x: cigar_len(x.PCIGAR), axis=1)
	if 'TYPE' in fetures_to_preprocess and ('ins' in set(x['TYPE']) or 'del' in set(x['TYPE'])) \
		and 'ALT' in fetures_to_preprocess and 'REF' in fetures_to_preprocess:
			x['INDEL_LEN'] = x.apply(indel_len, axis=1)
			x['INDEL_A_COUNTS'] = x.apply(lambda x: indel_base_count(x, 'A'), axis=1)
			x['INDEL_T_COUNTS'] = x.apply(lambda x: indel_base_count(x, 'T'), axis=1)
			x['INDEL_G_COUNTS'] = x.apply(lambda x: indel_base_count(x, 'G'), axis=1)
			x['INDEL_C_COUNTS'] = x.apply(lambda x: indel_base_count(x, 'C'), axis=1)
	if 'GT' in fetures_to_preprocess:
		x['GT'] = x['GT'].map({'0/0': 0, '0/1': 1, '1/1': 2}).astype(float)
	if 'FGT' in fetures_to_preprocess:
```

```
        x['FGT'] = x['FGT'].map({'0/0': 0, '0/1': 1, '1/1': 2}).astype(float)
    if 'MGT' in fetures_to_preprocess:
        x['MGT'] =  x['MGT'].map({'0/0': 0, '0/1': 1, '1/1': 2}).astype(float)
    if 't_GT' in fetures_to_preprocess:
        x['t_GT'] =  x['t_GT'].map({'0/0': 0, '0/1': 1, '1/1': 2}).astype(float)
    if 'TYPE' in fetures_to_preprocess:
        x['TYPE'] = x['TYPE'].map({'del': 0, 'ins' : 1, 'snp' : 2})
    if 'PTYPE' in fetures_to_preprocess:
        x['PTYPE'] = x['PTYPE'].map({'del': 0, 'ins' : 1, 'snp' : 2,\
                                                  'complex': 3, 'mnp': 4})
    if 'CIGAR' in fetures_to_preprocess:
        x['CIGAR'] = x['CIGAR'].astype("category",\
                                                    categories=len_alphabetic_category_order(x['CIGAR'])).cat.codes
    if 'PCIGAR' in fetures_to_preprocess:
        x['PCIGAR'] = x['PCIGAR'].astype("category",\
                                                    categories=len_alphabetic_category_order(x['PCIGAR'])).cat.codes
    if 'ALT' in fetures_to_preprocess:
        x['ALT'] = x['ALT'].astype("category",\
                                                    categories=len_alphabetic_category_order(x['ALT'])).cat.codes
    if 'REF' in fetures_to_preprocess:
        x['REF'] = x['REF'].astype("category",\
                                               categories=len_alphabetic_category_order(x['REF'])).cat.codes
    if 'CHROM' in fetures_to_preprocess:
        x['CHROM'] = x['CHROM'].map({'chr1': 0, 'chr2': 1, 'chr3': 2, 'chr4': 3, 'chr5': 4,\
                                                   'chr6': 5, 'chr7': 6, 'chr8': 7, 'chr9': 8, 'chr10': 9,\
                                                   'chr11': 10, 'chr12': 11, 'chr13': 12, 'chr14': 13, 'chr15': 14,\
                                                   'chr16': 15, 'chr17': 16, 'chr18': 17, 'chr19': 18, 'chr20': 19,\
                                                   'chr21': 20, 'chr22': 21}).astype(float)

    if remove_bad_values:
        bad_values_indexes = x[((x.MGT == 1.0) & (x.FGT == 2.0) & (x.t_GT == 0.0)) |\
                               ((x.MGT == 2.0) & (x.FGT == 1.0) & (x.t_GT == 0.0)) |\
                               ((x.MGT == 2.0) & (x.FGT == 2.0) & (x.t_GT != 2.0)) |\
                               ((x.MGT == 0.0) & (x.FGT == 0.0) & (x.t_GT != 0.0))].index
        x.drop(bad_values_indexes)
    return x



def roc_calibration_info(train_data, test_data, clf, family, color):
    p_train_data = preprocess_data(train_data)
    p_test_data = preprocess_data(test_data)
    x_train = p_train_data[ALL_FEATURES].values
    y_train = p_train_data[CLASS_COLUMN].values
    x_test = p_test_data[ALL_FEATURES].values
    y_test = p_test_data[CLASS_COLUMN].values
    bin_y_test =  label_binarize(y_test, classes=[0.0, 1.0, 2.0])
    n_classes = bin_y_test.shape[1]

    roc_curves_info = []
    calibration_plots_info = []
    new_calibration_plot_info = []
    y_true_y_prob_info = []

    for model in ('raw results', 'recalibrated'):
        if model == 'raw results':
            linestyle = '--'
            y_prediction = test_data['GT'].values
            y_score = np.array(test_data[['PP_homref', 'PP_het', 'PP_homalt']].reset_index(drop=True))
        else:
            linestyle = '-'
            clf.fit(x_train, y_train)
            y_prediction = clf.predict(x_test)
            if clf.__class__.__name__ != 'XGBClassifier':
                classifier = OneVsRestClassifier(clf)
            else:
```

```
                classifier = OneVsRestClassifier(xgb.sklearn.XGBClassifier(random_state=42, n_jobs=-1))
            classifier.fit(x_train, y_train)
            try:
                y_score = classifier.predict_proba(x_test)
            except:
                print("desicion function")
                y_score = classifier.desicion_function(x_test)

        accuraccy = accuracy_score(y_test, y_prediction)

        fpr = dict()
        tpr = dict()
        roc_auc = dict()
        for i in range(n_classes):
            fpr[i], tpr[i], _ = roc_curve(bin_y_test[:, i], y_score[:, i])
            roc_auc[i] = auc(fpr[i], tpr[i])

        # Compute micro-average ROC curve and ROC area
        fpr["micro"], tpr["micro"], _ = roc_curve(bin_y_test.ravel(), y_score.ravel())
        roc_auc["micro"] = auc(fpr["micro"], tpr["micro"])

        roc_label = family + ' ' + model + ' (AUC = %0.3f, ACC = %0.3f)' % (roc_auc["micro"], accuraccy)
        roc_curves_info.append([fpr["micro"], tpr["micro"], model, family, accuraccy,\
                                roc_auc["micro"], roc_label, linestyle, color])

        calib_label = family + ' ' + model
        y_true = np.array(y_test == y_prediction)
        y_prob = np.max(y_score, axis=1)

        #fraction_of_positives, mean_predicted_value = calibration_curve(y_true, y_prob, n_bins=15)
        calibration_plots_info.append([y_true, y_prob, model, family, calib_label, linestyle, color])
        new_calibration_plot_info.append([model, family, linestyle, color, y_test, y_prediction, y_score, y_prob])

    return(new_calibration_plot_info, roc_curves_info, calibration_plots_info)


def plot_roc_calibration(roc_curves_infos, calibration_plots_infos, settings):
    sns.set(color_codes=True)
    sns.set_style("white")
    sns.set_style("ticks")
    diagonal_line = 'k:'

    roc_curves_infos = [item for sublist in roc_curves_infos for item in sublist]
    calibration_plots_infos = [item for sublist in calibration_plots_infos for item in sublist]

    for r in roc_curves_infos:
        print("roc_info length", len(r))
        plt.plot(r[0], r[1],
                        lw=1, label=r[2],
                        linestyle=r[3], color = r[4])

        plt.plot([0, 1], [0, 1], diagonal_line)
        plt.xlim([0.0, 1.05])
        # plt.xlim([0.0, 0.01])
        plt.ylim([0.0, 1.05])
        plt.xlabel('False Positive Rate')
        plt.ylabel('True Positive Rate')
        plt.title('Receiver operating characteristic')
        plt.legend(loc="best")
    sns.despine()
    plt.savefig("/path/to/dir/{}_roc_curve".format(settings), transparent=True)

    plt.figure(figsize=(10, 8))
    ax1 = plt.subplot2grid((3, 1), (0, 0), rowspan=2)
    ax2 = plt.subplot2grid((3, 1), (2, 0))
```

```
        ax1.plot([0, 1], [0, 1], diagonal_line, label="Perfectly calibrated")
        for c in calibration_plots_infos:
                ax1.plot(c[1], c[0], label=c[3], linestyle = c[4], color = c[5])
                ax2.hist(c[2], range=(0, 1), bins=10, label=c[3], histtype="step", lw=1, linestyle=c[4], color=c[5])
                print(c[3] + ' #positions ' + str(len(c[2])))
                # sns.distplot(c[2], color=c[5], label = c[3], hist = False)
        ax1.set_ylabel("Fraction of positives")
        ax1.set_ylim([0.3, 1])
        ax1.legend(loc="best")
        ax1.set_title('Calibration plots (reliability curve)')
        ax1.set_xlim([0.3, 1])

        ax2.set_xlim([0.3, 1])
        ax2.set_xlabel("Mean predicted value")
        ax2.set_ylabel("Count")
        ax2.legend(loc="upper left", ncol=2)

        plt.tight_layout()
        sns.despine()
        plt.savefig("/path/to/dir/{}_calibration_plot.png".format(settings), transparent=True)


def main():
        """
        [test_family, Type(indel/snp), model, features, parameters, trained_on,\
         parents_test_settings, ml_acc, ml_auc, hoobari_acc, hoobari_auc]
        """
        colors = ['mediumturquoise', 'mediumorchid']
        roc_curves_results = {}
        calibration_plots_results = {}
        #for test_family in FAMILIES:
        for i, test_family in enumerate(TEST_TRAIN_FAMILIES):
                color = colors[i]
                train_families = TEST_TRAIN_FAMILIES[test_family]
                print("test family:", test_family)
                print("train families:", train_families)
                clf = CLASSIFIER
                for var_type in VAR_TYPES:
                        test_var_type_query = VAR_TYPES[var_type]
                        if var_type == 'indel':
                                train_var_type_query = ' in ("ins","del", "snp")'
                        else:
                                train_var_type_query = VAR_TYPES[var_type]
                        for test_option in TEST_OPTIONS:
                                test_query = TEST_OPTIONS[test_option]
                                parents_test_settings = test_option
                                if var_type == 'snp' and parents_test_settings == 'MhetFhom positions':
                                #and parents_test_settings in ('FhetMhom positions', 'MhetFhet positions'):
                                        train_query = '(MGT="0/1" or FGT="0/1")'
                                else:
                                        train_query = test_query
                                train_data = train_test_data_creator(train_families,\
                                                                             ALL_FEATURES + [CLASS_COLUMN],\
                                                                             train_var_type_query, train_query)
                                test_data = train_test_data_creator([test_family], ALL_FEATURES + [CLASS_COLUMN],\
                                                                                                     test_var_type_query, test_query)
                                family = test_family
                                #family = test_family.split("_")[0]
                                new_calibration_plot_info, roc_curves_info, calibration_plots_info = roc_calibration_info(train_data, test_data, clf, family, color)
                                pickle_name = "_".join([test_family, var_type, parents_test_settings])
                                pickle.dump((new_calibration_plot_info, roc_curves_info, calibration_plots_info),
open("/path/to/dir/{}_with_snpindel_train_on_test_setting".format(pickle_name), "wb"))

if __name__ == '__main__':
        main()
```
