# Supplementary material for: Bayesian-based noninvasive prenatal diagnosis of single-gene disorders
Source: Genome Res. 2019 Mar;29(3):428–38. doi: 10.1101/gr.235796.118 (PMC6396420; doi:10.1101/gr.235796.118)
Supplement: Supplemental Material [file supp_gr.235796.118_Supplemental_Methods.pdf]

Supplemental Methods

Processing of families from previous studies. Samples from families G1-4 were collected as described in the original studies (Chan et al. 2016; Kitzman et al. 2012).

Sample collection and DNA extraction. Samples from families E1-2 and G5 were collected during week 11 with informed consent. DNA from the CVS sample was extracted using the DNA Tissue protocol for the MagNA Pure Compact Nucleic Acid Isolation Kit I - Large Volume (Roche Life Science). Peripheral maternal blood was collected using 2-4 Ethylene-diamine-tetra-acetic acid (EDTA) tubes. Plasma was separated from blood by centrifugation at 4°C for 10 minutes at 1600 × g. The plasma was then centrifuged again at 16,000 × g for 10 minutes at room temperature to remove any residual cells. Extraction of cfDNA was performed using the QIAamp Circulating Nucleic Acid Kit (Qiagen). Removal of excess salts resulting from cfDNA purification was conducted using Agencourt AMPure XP beads (Beckman Coulter, Inc.) at a 2× ratio to cfDNA volume. Pure maternal DNA was extracted from leukocytes in the maternal buffy coats, using a routine protocol that includes (i) buffy coat separation and (ii) DNA purification using the Gentra Puregene Blood Kit (Qiagen) according to the manufacturer's instructions. Pure paternal DNA was collected and purified similarly.

Library preparation and sequencing. Library preparation for samples that underwent WGS was performed using the TruSeq DNA PCR-Free Library Prep Kit (Illumina) according to the manufacturer's instructions. This was followed by sequencing using NovaSeq (Illumina) with 150 paired-end reads for the cfDNA sample of family G5, and HiSeq X Ten System (Illumina) with 151-bp paired-end reads for the other WGS samples. For samples that underwent WES, library preparation was performed using the SureSelect V5 Exome Kit (Agilent) according to the manufacturer's instructions. Enrichment was achieved by hybridizing prepared genomic DNA to complementary RNA probe. Sequencing was then performed using HiSeq 4000 (Illumina) with 101-bp paired-end reads. Cell-free DNA samples were not

fragmented during library preparation, and were sequenced in two steps: (1) to a requested coverage of 50×, using HiSeq 4000 (Illumina) with 101-bp paired-end reads, and (2) to a requested coverage of 950×, using NovaSeq (Illumina) with 151-bp paired-end reads.

Alignment to the genome. Reads were aligned to the Genome Reference Consortium Human Build 37 (GRCh37/hg19) using Burrows-Wheeler v0.7.8 (Li and Durbin 2009) with default parameters, except for family G5, which was aligned to build 38 (GRCh38). As observed, this does not significantly affect the results, since we tested the model on millions of variants. The reference alleles for specific mutations that appear in the study were identical between the two builds. Duplicate reads, resulting from PCR clonality or optical duplicates, and reads mapping to multiple locations were excluded from downstream analysis.

Variant calling of pure genomic sequencing data. Single-nucleotide substitutions and small insertions and deletions were identified using Freebayes v1.1.0-3-g961e5f3 (Garrison and Marth 2012) using default parameters. Freebayes was first run on the aligned sequencing data of both parents together, then on the aligned data of the CVS sample using the variant sites that were identified in the parental genomes. Positions with depth >10 were, as well as parental positions with QUAL >30, were kept for downstream analysis.

Machine learning-based variant recalibration. A number of machine learning models were imported from the Scikit Learn (Pedregosa et al.), and were implemented using Python 3.5.1. Random forest algorithm was trained with a random state of 42 and 100 estimators. The exact settings are listed in Supplemental Fig. S7.
